# Phosphoproteomics identifies the DYRK1B protein kinase as a regulator of processing bodies

**DOI:** 10.1242/jcs.265054

**Published:** 2026-09-15

**Authors:** Anne L. Ashford, Suzan Ber, Miriam S. Ems, Emma Duncan, Kathryn Balmanno, Hannah Reeves, Rachael Huntly, Megan A. Cassidy, Harvey E. Johnston, David Oxley, Thaddeus Mutugi Nthiga, Terje Johansen, Marie Kluge, Ralf Jacob, Matthias Lauth, Simon J. Cook

**Affiliations:** ^1^ Signalling Programme, The Babraham Institute, Babraham Research Campus, Cambridge CB22 3AT, UK; ^2^ Philipps-University Marburg, Clinic of Gastroenterology, Endocrinology, and Metabolism, Center for Tumor and Immune Biology, Hans-Meerwein-Str. 3, 35043 Marburg, Germany; ^3^ Proteomics Facility, The Babraham Institute, Babraham Research Campus, Cambridge CB22 3AT, UK; ^4^ Molecular Cancer Research Group, Department of Medical Biology, Faculty of Health Sciences, UiT-The Arctic University of Norway, 9037 Tromsø, Norway; ^5^ Department of Molecular Cell Biology, Philipps-University Marburg, 35032 Marburg, Germany

**Keywords:** DYRK1B, Phosphoproteomics, Processing bodies, DCP1A, Condensates, RNA granules

## Abstract

Dual-specificity tyrosine-phosphorylation-regulated kinase 1B (DYRK1B) modulates the cell cycle and cell fate during development, and is deregulated in cancer and metabolic syndrome. However, only a few DYRK1B substrates have been defined, so we undertook a phosphoproteomics screen in cells that exhibit inducible DYRK1B expression. Motif analysis revealed enrichment for proline-directed serine or threonine phosphorylation sites (pSer–Pro or pThr–Pro), consistent with the consensus motif of class I DYRKs. Gene Ontology (GO) analysis revealed enrichment of proteins involved in mRNA binding, mRNA processing and ribonucleoprotein complexes. Several processing body (PB) components, including DCP1A, PATL1 (PAT1B), EDC3 and 4E-T (also known as EIF4ENIF1), were identified as DYRK1B-inducible phosphoproteins. DYRK1B also co-immunoprecipitated with DCP1A, PAT1B, EDC3, EDC4, DDX6 and XRN1. Super-resolution microscopy demonstrated that DYRK1B co-localised with DCP1A, DCP1B and DDX6 in PBs. Expression of DYRK1B increased PB abundance, whereas inhibition, depletion or knockout of DYRK1B reduced phosphorylation of DCP1A and 4E-T and decreased PB number. Re-expression of wild-type but not kinase-dead DYRK1B restored PB numbers in knockout cells. These findings reveal novel DYRK1B targets and establish DYRK1B as a regulator of PB abundance.

## Teaser

**Summary:** The kinase DYRK1B regulates processing body abundance and promotes phosphorylation of several processing body-associated proteins, revealing a new role for this kinase in cytoplasmic granule control.

## INTRODUCTION

The dual-specificity tyrosine-phosphorylation-regulated kinases (DYRKs) are found within the CMGC arm [encompassing cyclin-dependent kinases (CDKs), mitogen-activated protein kinases (MAPKs), glycogen synthase kinases (GSKs) and CDK-like kinases (CLKs)] of the eukaryote kinome and are highly conserved throughout evolution (Aranda et al., 2011; Becker et al., 1998; Becker and Joost, 1999; Han et al., 2012). DYRKs require phosphorylation of the second tyrosine in the activation loop tyrosine–X–tyrosine (Y–X–Y) motif to become mature, active kinases (Himpel et al., 2001; Lochhead et al., 2003). In contrast to most other protein kinases, this activating phosphorylation is not catalysed *in trans* by an upstream activating kinase but occurs during translation when DYRKs undergo intramolecular *cis*-autophosphorylation on tyrosine (Himpel et al., 2000; Lochhead et al., 2005). As a consequence, DYRKs are expressed in their active form, suggesting that regulation of their expression, localisation and/or protein–protein interactions are key for DYRK functions. DYRKs phosphorylate their substrates on serine or threonine residues, with a strong preference for a proline-directed context (pSer–Pro or pThr–Pro) (Himpel et al., 2000; Soundararajan et al., 2013) similarly to MAPKs and CDKs.

The mammalian DYRKs are divided into class I (DYRK1A and DYRK1B) and class II (DYRK2, DYRK3 and DYRK4) and have roles in transcription, mRNA splicing, cell cycle progression, survival and differentiation (Aranda et al., 2011; Becker and Joost, 1999). Both DYRK1A and DYRK1B can drive cell cycle arrest by phosphorylating and degrading cyclin D1 (CCND1) and increasing the abundance of the cyclin-dependent kinase inhibitors p21^CIP1^ (CDKN1A) and p27^KIP1^ (CDKN1B) (Ashford et al., 2014; Chen et al., 2013; Ewton et al., 2003; Soppa et al., 2014). Much of our knowledge of DYRKs has stemmed from study of *DYRK1A*, which is situated in the Down syndrome-critical region of chromosome 21, is overexpressed in foetal and adult brains of individuals with Down syndrome (Dowjat et al., 2007), and contributes to the clinical features of Down syndrome (Altafaj et al., 2001; Guimera et al., 1999; Smith et al., 1997). Indeed, triplication of *Dyrk1a* decreases nuclear CCND1 levels and drives cortical neurogenic defects in a mouse model of Down syndrome (Najas et al., 2015). Conversely, loss of a single copy of *Dyrk1a* in mice leads to increased apoptosis and decreased brain size (Fotaki et al., 2002), emphasising the importance of *Dyrk1a* gene dosage and activity. DYRK1B is implicated in several models of differentiation including myogenesis (Deng et al., 2003) and adipogenesis (Leder et al., 2003). Variants in *DYRK1B* have been reported in an inherited form of metabolic syndrome associated with early-onset coronary artery disease, obesity, hypertension and diabetes (Keramati et al., 2014). In addition, *DYRK1B* is amplified (Davis et al., 2013; Kuuselo et al., 2007) and mutated (Greenman et al., 2007) in certain cancers and can promote cell survival (Deng et al., 2009; Gao et al., 2009) and tumour immune evasion (Brichkina et al., 2024; Ems et al., 2025).

The preceding studies indicate that the DYRKs control critical cell fate decisions and are deregulated in disease, much like other CMGC kinases. Despite this, relatively few DYRK substrates have been reported that might account for their biological effects, whereas hundreds of substrates are known for the MAPKs ERK1 (MAPK3) and ERK2 (MAPK1) and the CDKs (Carlson et al., 2011; Courcelles et al., 2013; Malumbres, 2014; Petrone et al., 2016; Yoon and Seger, 2006). Many substrates have been reported for DYRK1A and include transcription factors (e.g. NFAT family members, GLI1), splicing factors [cyclin L2 (CCNL2), SF2 (SRSF1), SF3 (SF3B1)], a translation factor [eIF2Bε (EIF2B5)] and synaptic proteins [dynamin I (DNM1), synaptojanin-1 (SYNJ1)] (Park et al., 2009). DYRK1A also phosphorylates tau (MAPT) (Kimura et al., 2007) and this might be relevant to early-onset Alzheimer's disease in Down syndrome and a wider role for DYRK1A in neurodegeneration (Park et al., 2009). Some substrates are shared by different DYRKs; for example, both DYRK1A and DYRK1B phosphorylate CCND1 at T286 to target it for proteasomal degradation (Ashford et al., 2014; Chen et al., 2013; Soppa et al., 2014). Far fewer substrates have been defined for the class II DYRKs, although DYRK2 can phosphorylate p53 (TP53) at S46 (Taira et al., 2010). Beyond DYRK1A, other DYRK family members are increasingly recognised for their roles in neurodevelopmental diseases and cancer, making DYRKs potentially attractive therapeutic targets. Identifying new DYRK substrates can not only provide a molecular basis for DYRK biology, but also support drug discovery efforts, as validated substrates might serve as biomarkers of DYRK inhibition.

In addition to their established roles in cell cycle regulation, differentiation and metabolism, DYRK family kinases have been increasingly implicated in the control of membraneless organelles (Álvarez et al., 2003; Wippich et al., 2013). Membraneless organelles such as processing bodies (PBs), involved in translational repression and RNA turnover (Parker and Sheth, 2007), are dynamic ribonucleoprotein (RNP) assemblies formed through multivalent interactions between proteins and RNA and are regulated by post-translational modifications, including phosphorylation (Aizer et al., 2013; Chiang et al., 2013; Rzeczkowski et al., 2011).

In this study, we identify DYRK1B as a PB-associated kinase that directly phosphorylates the PB components DCP1A and 4E-T (EIF4ENIF1), promotes phosphorylation of additional PB-associated proteins, and localises to PBs. We further show that DYRK1B kinase activity is required to maintain PB abundance in multiple cell lines, including pancreatic cancer cells. Together, these findings establish DYRK1B as a novel regulator of PB abundance.

## RESULTS

### Identification of DYRK1B-induced phosphoproteins by phospho-SILAC mass spectrometry

**Fig. 1.**
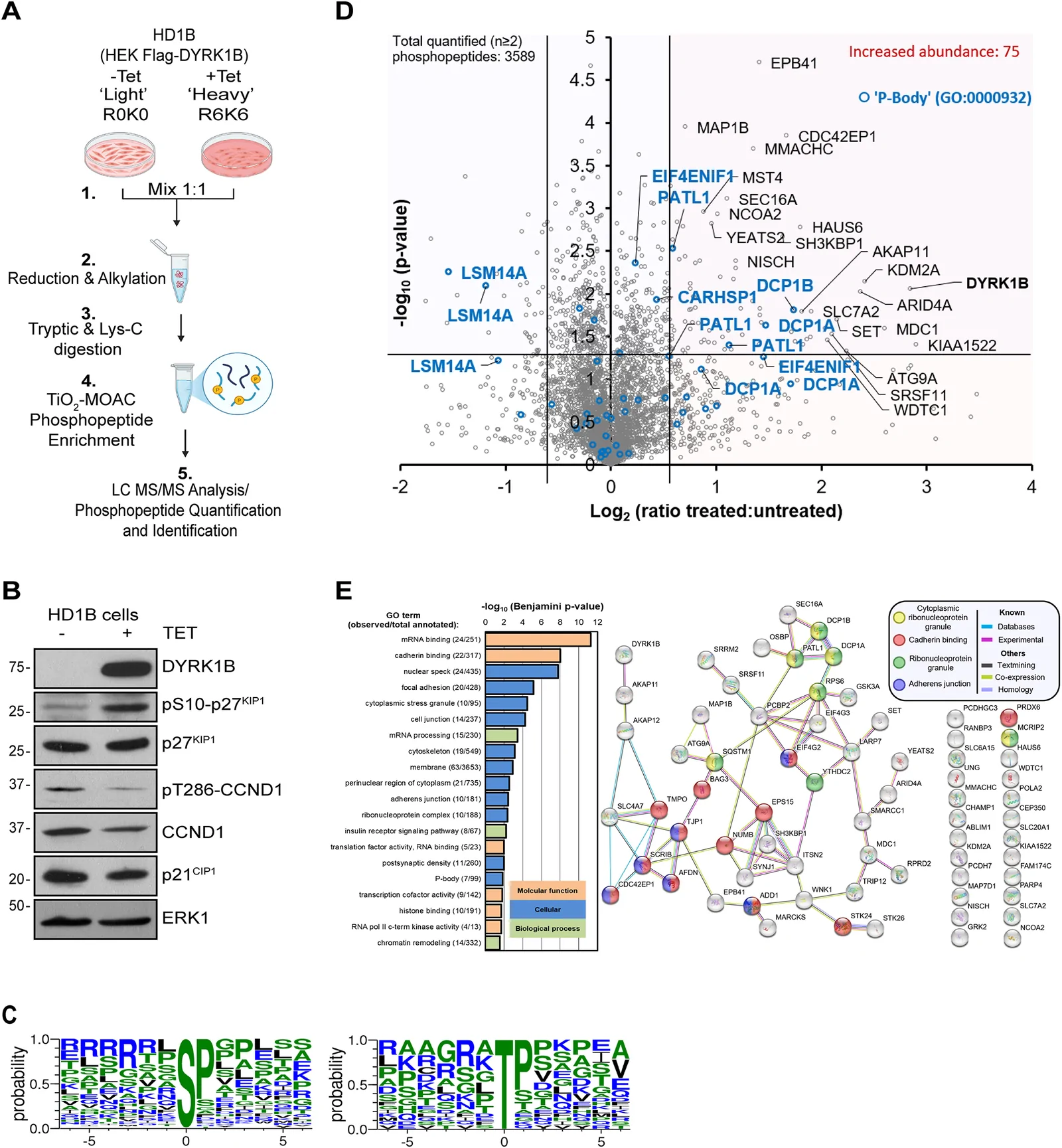
**Identification of DYRK1B-inducible phosphoproteins.** (A) Experimental workflow for phosphoproteomics by stable isotope labelling by amino acids in cell culture (phospho-SILAC) analysis of HEK293 cells with tetracycline (Tet)-inducible DYRK1B expression (HD1B cells). Equal amounts of lysates from untreated light (^12^C_6_-arginine and ^12^C_6_-lysine; R0K0)-labelled cells and tetracycline-treated heavy (^13^C_6_-arginine and ^13^C_6_-lysine; R6K6)-labelled cells were combined and digested, and the phosphopeptides were enriched and analysed by liquid chromatography (LC) coupled with tandem mass spectrometry (MS/MS). TiO_2_-MOAC, Titanium dioxide metal oxide affinity chromatography. Created in BioRender by Ber, S. 2026. https://BioRender.com/48tdfa8. This figure was sublicensed under CC-BY 4.0 terms. (B) Immunoblot validation of DYRK1B induction after tetracycline treatment (1 μM, 8 h), with increased p27 phosphorylation and reduced cyclin D1 (CCND1) levels. *n*=3 independent experiments. (C) Motif analysis of differentially phosphorylated peptides following DYRK1B induction. Motif analysis was performed using WebLogo. Amino acids are coloured according to biochemical properties: polar amino acids are in green, basic amino acids in blue, acidic amino acids in red and hydrophobic amino acids in black. (D) Volcano plot detailing SILAC-quantified phosphopeptide abundance changes upon treatment. The red shaded region indicates significantly increased phosphopeptides (>1.5-fold, *P*<0.05). Proteins annotated with the term ‘P-Body’ (processing body or PB, GO:0000932) are shown in blue. See also Table S1. (E) Functional characterisation of proteins with increased phosphorylation (>1.5-fold, *P*<0.05). Gene Ontology (GO) enrichment analysis (left) identifies significantly overrepresented biological processes. STRING network analysis of the same proteins (right) reveals interaction networks and functional clustering of DYRK1B-regulated proteins.

The DYRKs undergo *cis*-autophosphorylation at a conserved tyrosine in their activation loop during translation, so they are active once they are expressed. Consequently, we used HEK293 cells with tetracycline-inducible expression of DYRK1B (HD1B cells) (Ashford et al., 2014) to identify DYRK1B-inducible phosphoproteins using stable isotope labelling by amino acids in cell culture (SILAC). HD1B cells were grown in light (^12^C_6_-arginine and ^12^C_6_-lysine; R0K0) or heavy (^13^C_6_-arginine and ^13^C_6_-lysine; R6K6) SILAC medium for 11 days, by which time ^13^C incorporation in cells grown in R6K6 medium was 98–99%. R6K6-labelled cells were then treated with tetracycline for a further 8 h prior to lysis (Fig. 1A). This timepoint was chosen as the earliest point at which DYRK1B expression was maximal and was a trade-off; earlier timepoints would more likely identify proximal DYRK1B targets (e.g. direct DYRK1B substrates), whereas longer timepoints would more likely identify distal phosphorylation events (e.g. those arising from activation of other DYRK1B-dependent protein kinases). An aliquot of each dish was set aside for western blot analysis and confirmed expression of DYRK1B, turnover of CCND1 and phosphorylation of p27^KIP1^ (Fig. 1B) (Ashford et al., 2014). The remaining lysates were normalised for protein content, pooled as control light-labelled and DYRK1B-induced heavy-labelled pairs, reduced and digested with trypsin. Phosphopeptides were enriched using titanium dioxide beads and analysed by liquid chromatography (LC) coupled with tandem mass spectrometry (MS-MS) (Fig. 1A). The full results of this are shown in Table S1. An example trace for a differentially phosphorylated peptide is shown in Fig. S1; this peptide was identified as being derived from DCP1A, a regulatory subunit that binds to the catalytic subunit DCP2 to form a mRNA decapping complex (Mugridge et al., 2016).

All differentially phosphorylated peptides identified were phosphorylated on serine or threonine, except for one DYRK1B-derived peptide containing the tyrosine-phosphorylated activation loop sequence IYQYIQSR. This peptide increased in abundance following tetracycline treatment (Table S1), consistent with inducible expression of DYRK1B. Motif analysis derived from all differentially phosphorylated peptides revealed a strong enrichment for proline-directed sites (Ser–Pro or Thr–Pro) with an additional preference for arginine upstream of the phosphoacceptor site (Fig. 1C). This agrees with previous reports of class I DYRK substrate selectivity (Campbell and Proud, 2002; Himpel et al., 2000), suggesting that some of the proteins identified are direct DYRK1B substrates.

The DYRK1B-inducible phosphoproteins that we identified (Fig. 1D) are involved in a range of biochemical processes, including: cell signalling [ADRBK1 (GRK2), AKAP11, GSK3A, NISCH, SCRIB, STK24 (MST3), STK26 (MST4), SYNJ1, WNK1]; protein trafficking (EPS15, SH3KBP1); autophagy and proteostasis [ATG9A, BAG3, SQSTM1 (p62)]; protein ubiquitylation (TRIP12, WDTC1); protein synthesis (RPS6, EIF4G2, EIF4G3); the actin cytoskeleton (ABLIM1, CDC42EP1); cell–cell adhesion [afadin (AFDN)]; microtubule dynamics (KATNA1); metabolite or amino acid transport (SLC4A7, SLC6A15, SLC7A2, SLC20A1); the DNA damage checkpoint (MDC1); histone modifications and chromatin remodelling (KDM2A, SET, SMARCC1); regulation of transcription (ARID4A); and the cell cycle (MPLKIP, RBL1). Some of these proteins have been reported to be phosphorylated upon DYRK1B overexpression (Dong et al., 2020), reinforcing the robustness of our observations. Notably, GO analysis of DYRK1B-inducible phosphoproteins suggested roles in mRNA binding, mRNA processing and RNP complexes, as well as roles in focal adhesions, cadherin binding and adherent junctions (Fig. 1E).

### Validation of PB proteins as DYRK1B targets

Prompted by the GO analysis, we noted that DCP1A, DCP1B, 4E-T, PAT1B (also known as PATL1) and EDC3 were phosphorylated upon DYRK1B expression (Table S1). These proteins have roles in mRNA decapping (DCP1A, DCP1B, EDC3, PAT1B) or translational repression (4E-T) and localise together in cytosolic membraneless organelles or condensates called PBs. Notably, many of the DYRK1B-driven phosphorylation sites on DCP1A, DCP1B, 4E-T and PAT1B identified in the screen were proline directed (Table S1), agreeing with the DYRK phosphorylation site consensus.

**Fig. 2.**
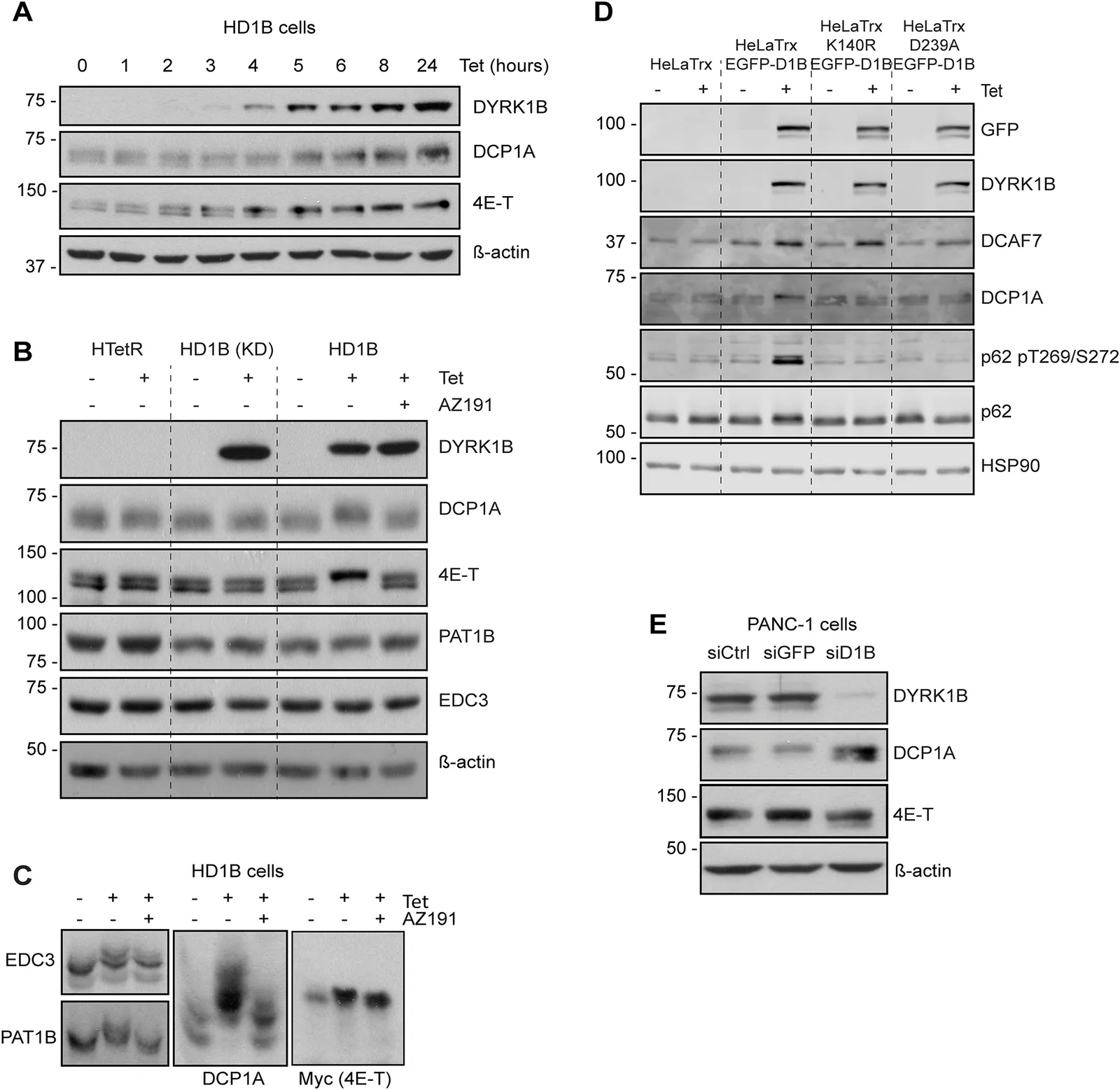
**Validation of phospho-SILAC targets.** (A) Time-course induction of FLAG–DYRK1B in HD1B cells using tetracycline (Tet; 1 μM) for up to 24 h showed increased phosphorylation of DCP1A and 4E-T targets. Phosphorylation coincides with the expression of DYRK1B protein. *n*=3 independent experiments. (B) Expression of wild-type but not kinase-dead (KD) DYRK1B, upon tetracycline treatment (1 μM) for 24 h, resulted in phosphorylation of DCP1A, 4E-T and PAT1B in HD1B cells. This phosphorylation was inhibited when cells were treated with the DYRK1 inhibitor AZ191 (1 μM). *n*=3 independent experiments. (C) Phos-tag gel showing phosphorylation-dependent shifts of DCP1A, PAT1B, EDC3 and ectopically expressed Myc-tagged 4E-T upon DYRK1B expression, and reduced phosphorylation following treatment with AZ191 (1 μM). *n*=3 independent experiments. (D) Expression of wild-type but not kinase-dead (K140R and D239A mutants) EGFP–DYRK1B in HeLa Flp-In T-REx cells increased phosphorylation of DCP1A and p62 (T269/S272). *n*=3 independent experiments. (E) Knockdown of DYRK1B in PANC-1 cells using siRNA reduced DCP1A and 4E-T phosphorylation. *n*=3 independent experiments.

In follow-up experiments, inducible DYRK1B expression led to a time-dependent reduction in electrophoretic mobility of DCP1A and 4E-T in HD1B cells. This shift is typical of phosphorylation and correlated well with the onset of DYRK1B expression (Fig. 2A). The DYRK1B-induced mobility shift of DCP1A and 4E-T was reversed by AZ191, a class I DYRK inhibitor with some selectivity for DYRK1B over DYRK1A (Ashford et al., 2014), whereas a catalytically inactive or kinase-dead DYRK1B D239A mutant failed to induce mobility shift of DCP1A or 4E-T (Fig. 2B). Together, these results indicate that the DCP1A and 4E-T gel shifts reflect DYRK1B-driven phosphorylation in cells. On conventional SDS-PAGE gels, there were very subtle changes for PAT1B and EDC3, whereas Phos-tag gels revealed clear DYRK1B-dependent phosphorylation of PAT1B and EDC3 and enhanced the phosphorylation-dependent gel shift of DCP1A and 4E-T (Fig. 2C). DYRK1B-dependent phosphorylation of DCP1A was also observed in HeLa cells expressing DYRK1B from the T-REx system (HeLa Flp-In T-REx), which showed a DCP1A mobility shift and increased phosphorylation of the autophagy cargo receptor p62 (SQSTM1) at T269/S272, confirming the phospho-SILAC data (Fig. 2D). In both cases, these phosphorylation events were not observed upon expression of either of two kinase-dead mutants, DYRK1B K140R or DYRK1B D239A. DYRK1B expression increased the abundance of DCAF7, a scaffold protein that can recruit class I DYRKs to some of their substrates (Glenewinkel et al., 2016; Yu et al., 2019). This increase in DCAF7 was also observed with DYRK1B K140R or DYRK1B D239A, suggesting that DCAF7 is stabilised by its interaction with DYRK1B, independent of DYRK1B kinase activity.

We have demonstrated that DCP1A, PAT1B, EDC3 and 4E-T underwent phosphorylation upon expression of DYRK1B in cells. To see whether DYRK1B was required for their phosphorylation, we analysed pancreatic cancer cell lines as *DYRK1B* amplifications are associated with ∼7% of pancreatic cancer (Brichkina et al., 2024) (Fig. S2A). PANC-1 cells harbour a 19q13.1 amplification that includes the *DYRK1B* gene and exhibit greatly elevated expression of DYRK1B relative to that in BxPC3 cells, which lack the 19q13.1 amplification (Fig. S2B). When PANC-1 cells were transfected with siRNA to knock down DYRK1B expression, DCP1A and 4E-T mobility on SDS-PAGE increased, consistent with their dephosphorylation (Fig. 2E). Thus, in PANC-1 cells, phosphorylation of these PB proteins was dependent upon endogenous DYRK1B.

### DCP1A and 4E-T are direct substrates of DYRK1B

**Fig. 3.**
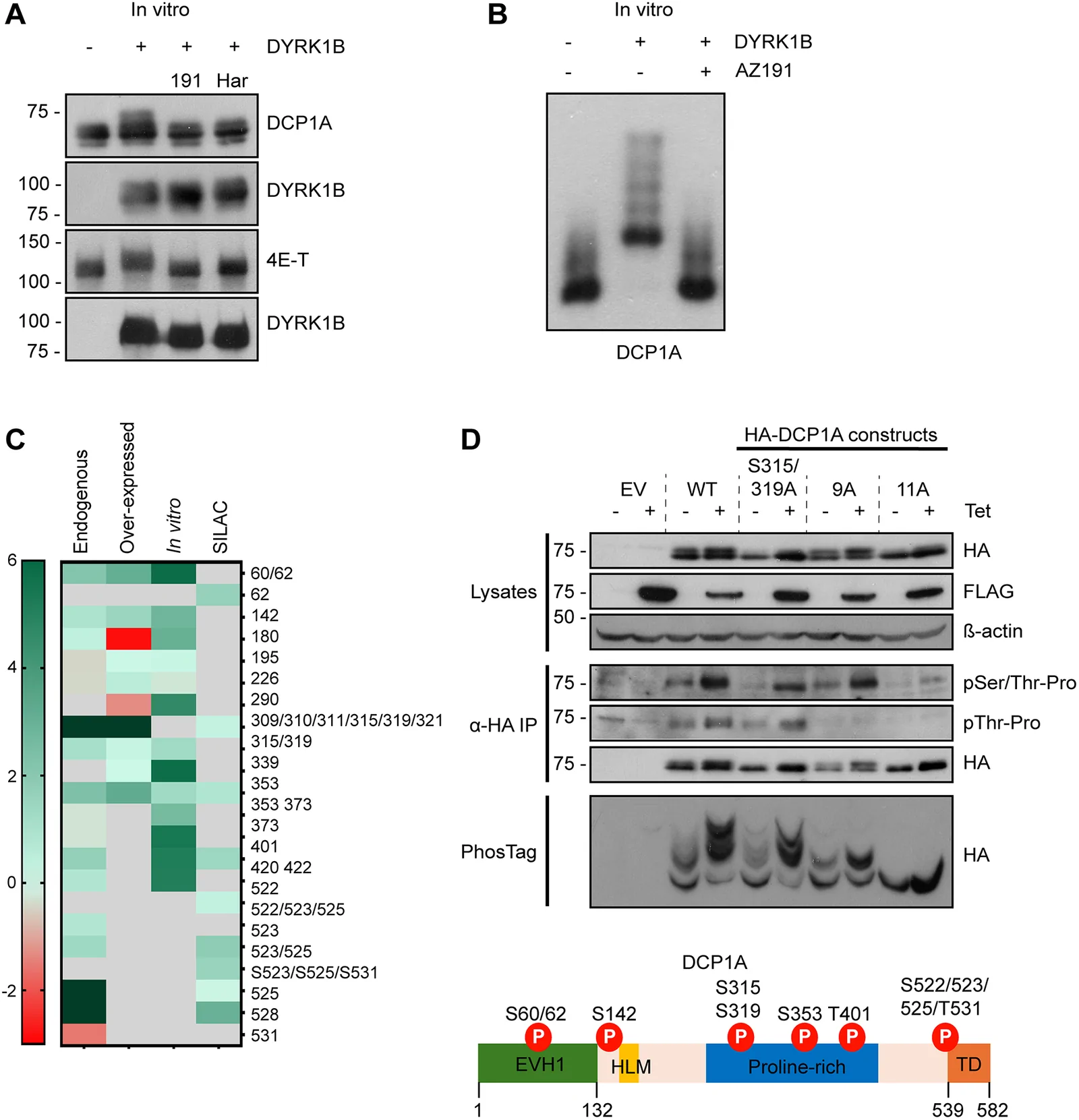
**DCP1A and 4E-T are direct substrates of DYRK1B.** (A) *In vitro* kinase assay showing phosphorylation of DCP1A and 4E-T upon addition of recombinant DYRK1B and reduced phosphorylation when co-treated with the class 1 DYRK inhibitors AZ191 (1 μM) or harmine (1 μM). *n*=3 independent experiments. (B) Phos-tag gel showing clear shift of *in vitro*-phosphorylated DCP1A protein upon DYRK1B addition and the dephosphorylation upon co-treatment with AZ191 (1 μM). *n*=3 independent experiments. (C) Heatmap showing log_2_-transformed changes in DCP1A phosphosite abundance upon DYRK1B expression across multiple experimental conditions: DYRK1B-inducible phosphorylation of endogenous DCP1A in HD1B cells; DYRK1B-inducible phosphorylation of overexpressed DCP1A in HD1B cells; direct *in vitro* phosphorylation by recombinant DYRK1B; and DCP1A phosphorylation sites identified by phospho-SILAC. Values represent log_2_ fold change (FC) relative to control for each condition. Grey boxes indicate missing values, corresponding to phosphosites not detected or not quantified in the indicated condition. Darkest green indicates log_2_FC values >6, which are displayed at the maximum colour intensity to improve visualisation of lower-magnitude phosphorylation changes. (D) Immunoprecipitation (IP) of wild-type (WT) and mutant HA–DCP1A (S315A/S319A, 9A and 11A phospho-null mutants) from HD1B-transfected cells, validating multisite phosphorylation sites in DCP1A upon DYRK1B induction. *n*=3 independent experiments. The schematic below summarises the identified phosphorylation sites in DCP1A. HLM, helical-leucine motif; TD, trimerisation domain.

Phos-tag gels indicated that DCP1A underwent multisite phosphorylation following DYRK1B expression in cells. To assess whether DCP1A and 4E-T were direct substrates of DYRK1B, we conducted *in vitro* kinase assays with purified components. Purified, recombinant DYRK1B catalysed phosphorylation of both DCP1A and 4E-T *in vitro* and this was inhibited by the inclusion of AZ191 and harmine, a DYRK1A inhibitor with some activity against DYRK1B (Göckler et al., 2009) (Fig. 3A). Phos-tag analysis of these reactions revealed multiple phosphorylated species of DCP1A, consistent with multisite modification (Fig. 3B).

To define DYRK1B-dependent phosphorylation sites in DCP1A, we used targeted mass spectrometry of DCP1A with tetracycline-inducible DYRK1B in HD1B cells. Fig. 3C shows sites of direct *in vitro* DCP1A phosphorylation by recombinant DYRK1B, phosphorylation sites in overexpressed DCP1A in tetracycline-induced HD1B cells, and phosphorylation sites in endogenous DCP1A in tetracycline-induced HD1B cells (also shown in detail in Table S2). Among these DYRK1B-inducible phosphorylation sites, S315 and S319 were previously proposed to be phosphorylated by the proline-directed kinases ERK1/2 (Chiang et al., 2013) and JNK (Rzeczkowski et al., 2011), and to be phosphorylated separately during mitosis, although the kinase responsible was not defined (Aizer et al., 2013).

To assess the contribution of these sites, we generated a S315A and S319A double mutant and analysed phosphorylation by mobility shift and phospho-specific antibodies. Mutation of these residues reduced but did not abolish the DYRK1B-dependent gel shift and decreased reactivity with the pSer/Thr–Pro antibody, indicating that S315 and S319 are DYRK1B targets in cells (Fig. 3D). To further evaluate additional sites, we generated a mutant in which nine candidate residues identified by mass spectrometry were substituted with alanine (9A). This mutant showed a marked reduction in DYRK1B-dependent gel shift and loss of pThr–Pro reactivity. Combining these mutations with S315A and S319A (11A mutant) further suppressed phosphorylation, although a residual signal remained, suggesting the presence of additional DYRK1B-dependent sites. The residual gel shift and reactivity with the pSer/Thr–Pro antibody suggests that there are other as yet unidentified DYRK1B phosphorylation sites in DCP1A. These phosphorylation sites are distributed across functionally defined regions of DCP1A, including the N-terminal EVH1 domain (S62), which is known to mediate interactions with DCP2, the central proline-rich region (S315, S319, S353, T401 and S422), the evolutionarily conserved motif I (S142 and S180) and the C-terminal trimerisation domain (S525), indicating that DYRK1B targets multiple structural modules of the protein. These results suggest that S62, S315/S319, S353, T401 and S525 are high-confidence DYRK1B phosphorylation sites in cells, whereas S142, S180 and S422 are possible DYRK1B phosphorylation sites, all of which are proline directed.

Collectively, these data identify DCP1A and 4E-T as direct DYRK1B substrates, establish DCP1A as a multisite target and show that DYRK1B promotes phosphorylation of multiple PB-associated proteins.

### DYRK1B associates with its substrates and co-localises with them in PBs

**Fig. 4.**
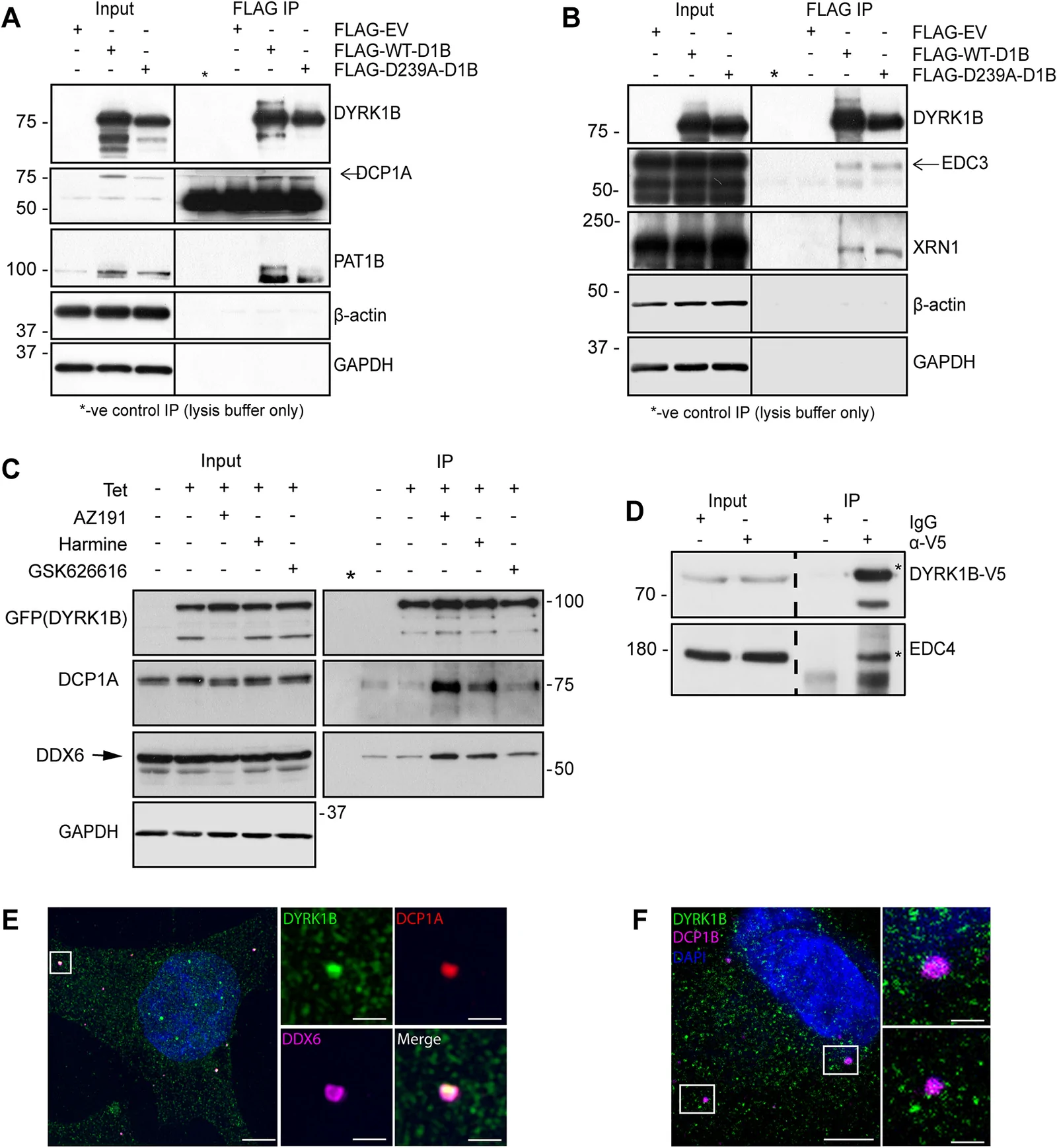
**PB components interact with and co-localise with DYRK1B in PBs.** (A) Immunoprecipitation analysis of FLAG-tagged wild-type (WT) or kinase-dead (D239A) DYRK1B (D1B) from transfected HeLa cells showed that it associates with endogenous DCP1A and PAT1B. *n*=3 independent experiments. (B) Immunoprecipitation analysis of FLAG-tagged WT or kinase-dead (D239A) DYRK1B from transfected HeLa cells showed that it associates with endogenous EDC3 and XRN1. *n*=3 independent experiments. (C) Expression of EGFP-tagged WT DYRK1B was induced in HeLa Flp-In T-REx cytosolic cell extracts by tetracycline (1 μM) treatment for 24 h and cells were consecutively treated with DYRK inhibitors AZ191 (1 μM), harmine (1 μM) and GSK626616 (1 μM) for 8 h. Immunoprecipitation analysis of EGFP–DYRK1B showed interaction with the endogenous PB proteins DCP1A and DDX6. *n*=3 independent experiments. (D) Immunoprecipitation analysis of stably overexpressed V5-tagged DYRK1B in PaTu 8988T cells showed interaction with the PB component EDC4. *n*=3 independent experiments. (E) High-resolution (structured illumination microscopy) image of HeLa Flp-In T-REx cells expressing EGFP–DYRK1B, showing co-localisation with mRNA-decapping proteins DCP1A (red) and DDX6 (magenta). *n*=3 independent experiments. (F) Super-resolution (stimulated emission depletion microscopy) image of endogenous DYRK1B (green) co-localising with the PB component DCP1B (magenta) in PANC-1 cells. *n*=3 independent experiments. Scale bars: 5 μm; 1 μm (zoomed panels).

Having identified DCP1A, PAT1B, EDC3 and 4E-T as DYRK1B-inducible phosphoproteins, we next examined whether these proteins physically associate with DYRK1B. FLAG-tagged wild-type and kinase-dead (D239A) DYRK1B were transiently expressed and subjected to FLAG- or GFP-based immunoprecipitation in both HeLa and HEK293 cells. Across both systems, DYRK1B co-immunoprecipitated with multiple PB components, including endogenous DCP1A, PAT1B and EDC3 in HeLa cells (Fig. 4A,B), as well as with ectopically expressed HA–DCP1A and EGFP–PAT1B in HEK293 cells (Fig. S3A,B). In addition, DYRK1B associated with endogenous XRN1, a key 5′–3′ exoribonuclease involved in mRNA decay, in both cell lines (Fig. 4B; Fig. S3C), and with the RNA helicase DDX6, which functions in translational repression and RNA turnover, in HeLa cells (Fig. 4C).

These interactions were retained with the kinase-dead DYRK1B D239A mutant, indicating that binding of DYRK1B to PB components does not require its catalytic activity (Fig. 4A,B; Fig. S3A–C). To further assess the role of kinase activity in complex formation, HeLa cells expressing EGFP–DYRK1B were treated with DYRK inhibitors prior to immunoprecipitation. Pharmacological DYRK inhibition increased the amount of DCP1A and DDX6 co-immunoprecipitating with DYRK1B, with the DYRK1 inhibitor AZ191 showing the strongest effect followed by harmine, whereas the class II DYRK inhibitor GSK626616 had a weaker impact (Fig. 4C). These findings suggest that acute inhibition of DYRK1B activity can stabilise or alter its association with PB components. Consistent with these observations, DYRK1B also co-immunoprecipitated with the PB component EDC4 in the pancreatic cancer cell line PaTu 8988T expressing V5-tagged DYRK1B (Fig. 4D), supporting a broader association of DYRK1B with the PB machinery.

To determine whether DYRK1B localises to PBs, we used both super-resolution and widefield microscopy to examine its subcellular distribution. DYRK1B localisation was assessed either by imaging EGFP-tagged DYRK1B in HeLa Flp-In T-REx cells or by staining endogenous DYRK1B in two pancreatic cancer cell lines, PANC-1 and PaTu 8988T. PBs were identified using co-localised DCP1A and DDX6 foci in HeLa cells, whereas DCP1B served as the PB marker in pancreatic cancer cells. These are all well-established PB components that label overlapping PB structures and, therefore, provide comparable measures of PB abundance. In all cell types examined, DYRK1B co-localised with the established PB markers DCP1A, DCP1B and DDX6 in cytoplasmic puncta characteristic of PBs (Fig. 4E,F; Fig. S3D). In addition, live-cell imaging of PaTu 8988T cells expressing DYRK1B–EGFP revealed cytoplasmic GFP puncta that co-localised with mCherry–DDX6 and showed similar dynamic behaviour, consistent with PBs (Movie 1).

We next compared the localisation of different DYRK family members. EGFP–DYRK1A and EGFP–DYRK1B both showed co-localisation with the PB markers DCP1A and DDX6, whereas EGFP–DYRK2 and EGFP–DYRK3 displayed only weak co-localisation (Fig. S3F). In parallel, overexpression of DYRK1A and DYRK1B produced modest mobility changes in DCP1A and 4E-T, consistent with increased phosphorylation of these PB proteins, whereas effects on PAT1B and EDC3 were less apparent under these conditions (Fig. S3E). Together, these observations suggest that DYRK1A and DYRK1B show the clearest association with PB components in this overexpression system, although these data do not exclude potential contributions from DYRK2 or DYRK3 in other contexts.

### DYRK1B regulates PB abundance

**Fig. 5.**
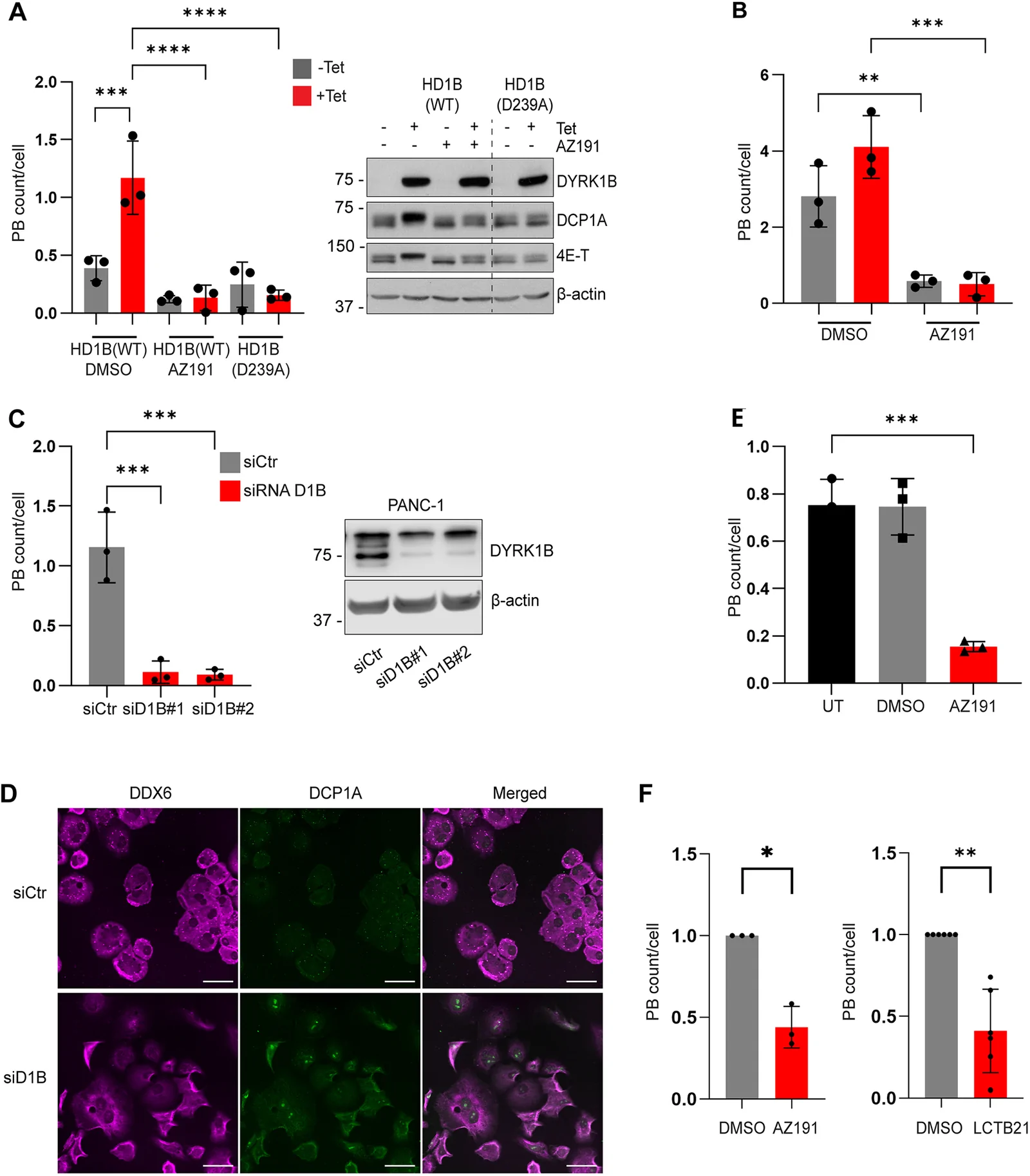
**DYRK1B regulates PB abundance.** (A,B) High-content quantification of PBs (defined as DCP1A and DDX6 double-positive foci) in HD1B cells (A) and HeLa Flp-In T-REx EGFP–DYRK1B cells (B). Induction of wild-type (WT) but not kinase-dead (D239A) DYRK1B with tetracycline (1 μM, 24 h) increased PB number, which was reduced by AZ191 (1 μM). Representative immunoblots show DYRK1B expression and the mobility shifts of DCP1A and 4E-T, with β-actin as a loading control (A) (*n*=3). (C) High-content quantification of PBs in PANC-1 cells transfected with two different siRNAs against DYRK1B, showing reduction in PB number. Representative immunoblot confirms DYRK1B knockdown by both siRNAs, with β-actin as a loading control (*n*=3). (D) Representative images of PANC-1 cells used for the quantification in C, stained for DCP1A (green) and DDX6 (magenta). PBs were quantified as discrete cytoplasmic foci positive for both DCP1A and DDX6. DYRK1B depletion strongly reduced the number of DCP1A and DDX6 double-positive cytoplasmic foci. Scale bars: 10 μm. (E) High-content quantification of PBs in PANC-1 cells treated with the DYRK1 inhibitor AZ191 (1 μM) showed reduction in PB number. (F) Quantification of PBs using DCP1B as marker showed reduced PB number after 24 h of treatment with the DYRK1 inhibitors AZ191 (1 μM) (left, *n*=3) and LCTB21 (1 μM) (right, *n*=6) in PANC-1 cells. Data are presented as mean±s.d. of three independent experiments, unless otherwise stated. *P*-values were calculated using one-way ANOVA (A,B,C,E) with Dunnett's multiple comparisons test, and with one-sample unpaired two-tailed *t*-test (F). *P≤0.05; **P≤0.01; ***P≤0.001; ****P≤0.0001.

**Fig. 6.**
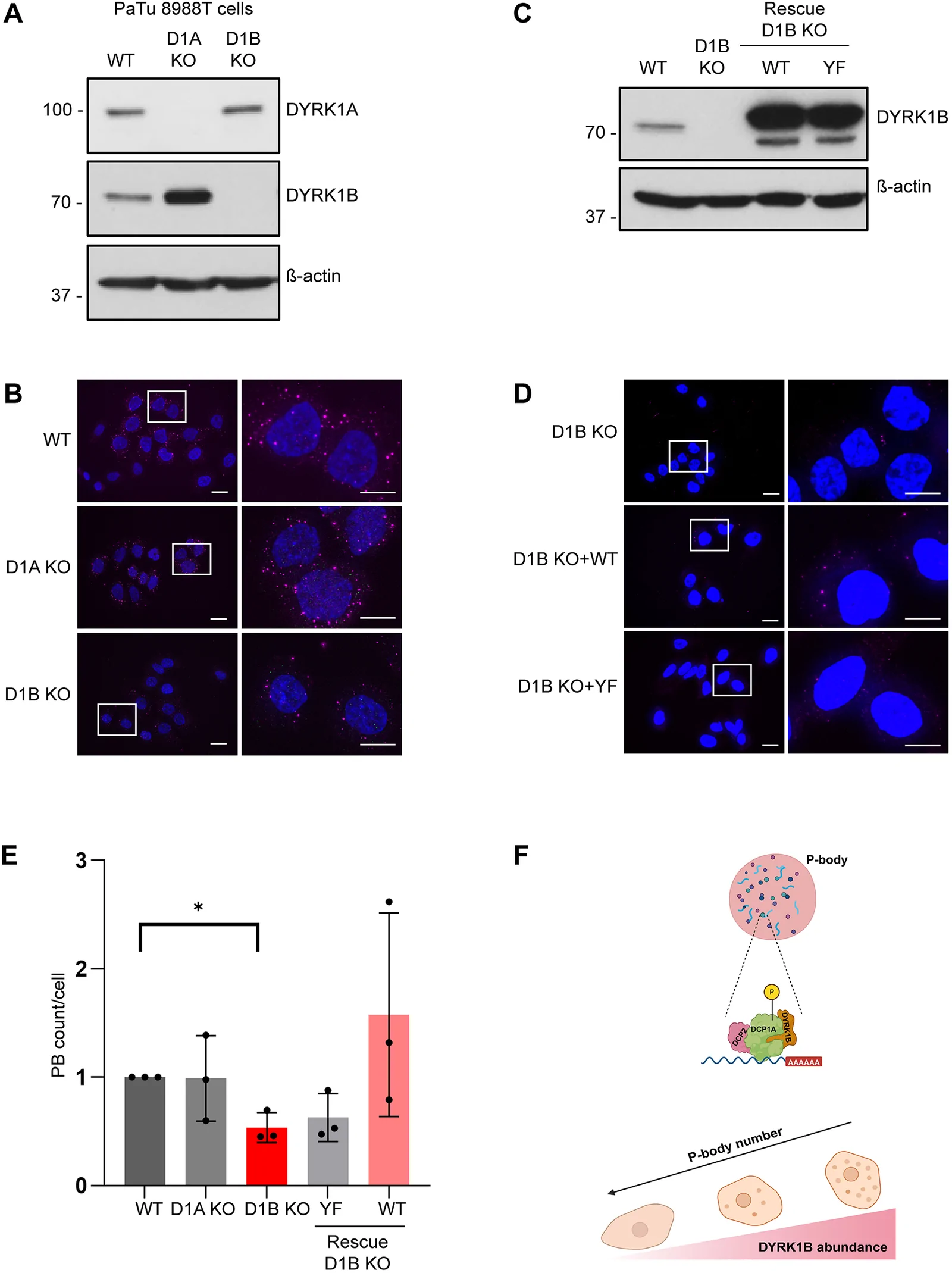
**DYRK1B controls PB abundance in pancreatic cancer cells.** (A) Representative western blot images showing loss of DYRK1A (D1A KO) or DYRK1B (D1B KO) in PaTu 8988T pancreatic cancer cells. *n*=3 independent experiments. (B) Representative images of DCP1B foci (magenta) in D1A KO and D1B KO PaTu 8988T cells. The loss of DYRK1B, but not of DYRK1A, leads to a reduction in PB number. Scale bars: 20 μm (whole-cell images); 10 μm (zoomed panels). (C) Representative western blot images showing rescue of DYRK1B KO using DYRK1B WT and kinase-dead DYRK1B (YF; Y271F/Y273F) in PaTu 8988T cells. *n*=3 independent experiments. (D) Representative images of DCP1B foci (magenta) in D1B KO PaTu 8988T cells rescued with either DYRK1B WT or kinase-dead DYRK1B (YF). Note the increase of DCP1B foci after rescue with DYRK1B WT but not with DYRK1B YF. Scale bars: 20 μm (whole-cell images); 10 μm (zoomed panels). (E) Quantification of DCP1B foci depicted in panels B and D. Data represent mean±s.d. of three independent experiments; *P*-value is calculated using unpaired two-tailed *t*-test. *P≤0.05. (F) Model showing previously unknown association of DYRK1B with PBs. DYRK1B localises to PBs, associates with several PB components (DCP1A, EDC3, EDC4, PAT1B, XRN1, DDX6) and promotes phosphorylation of these PB-associated proteins. DYRK1B kinase activity is required to maintain PB abundance. Created in BioRender by Ber, S. 2026. https://BioRender.com/9vtsnfr. This figure was sublicensed under CC-BY 4.0 terms.

Considering the data so far, we investigated whether DYRK1B regulates PB abundance. Upon quantifying PBs (defined as DCP1A- and DDX6-positive cytoplasmic foci) in HD1B cells with high-content imaging, we found that expression of DYRK1B in HD1B cells caused a 3-fold increase in PB abundance that was reversed by AZ191 treatment (Fig. 5A). In contrast, expression of kinase-dead DYRK1B D239A in HD1B cells failed to increase PB abundance (Fig. 5A). These studies were extended to HeLa Flp-In T-REx EGFP–DYRK1B cells, in which the basal abundance of PBs was higher and DYRK1B expression caused only a modest increase in PB abundance (Fig. 5B). However, AZ191 treatment decreased both basal and tetracycline-induced PB abundance, suggesting that the higher basal abundance of PBs was in part driven by DYRK1B. These results indicate that DYRK1B expression drives an increase in PB abundance in a kinase-dependent manner (Fig. 5B). As AZ191 might also inhibit DYRK1A at this concentration, this result supports a role for class I DYRK activity but does not distinguish DYRK1B from DYRK1A. We therefore used genetic depletion and knockout-rescue approaches to define the DYRK1B-specific contribution (Fig. 6).

To address whether DYRK1B was required to maintain PB number in a clinically relevant setting, we used the pancreatic cancer cell line PANC-1. siRNA-mediated knockdown of DYRK1B with two different siRNAs in PANC-1 cells, in which DYRK1B is amplified (Fig. S2B), caused a striking >70% reduction in PB abundance as assessed by high-content imaging (Fig. 5C,D). This reduction in PB number was phenocopied by treatment with the class I DYRK inhibitor AZ191 (Fig. 5E) as well as LCTB21 (a DYRK1 inhibitor currently in phase 1 clinical trials; Meijer et al., 2024) (Fig. 5F).

Finally, we used CRISPR/Cas9 gene editing to generate PaTu 8988T cells with knockout of DYRK1A or DYRK1B (Fig. 6A). Interestingly, DYRK1B expression was increased in DYRK1A knockout cells, consistent with previous reports that DYRK1A depletion can induce compensatory upregulation of DYRK1B, whereas reciprocal upregulation of DYRK1A following DYRK1B depletion is not consistently observed (Ackeifi et al., 2020; Pastor et al., 2024). Under these conditions, DYRK1B knockout significantly reduced PB number in PaTu 8988T cells, whereas DYRK1A knockout did not (Fig. 6B,E). Re-expression of wild-type DYRK1B, but not kinase-inactive DYRK1B (Y271F/Y273F) restored PB abundance, demonstrating that DYRK1B kinase activity is required for this effect (Fig. 6C–E). These data support a requirement for DYRK1B kinase activity in maintaining PB abundance in pancreatic cancer cells.

In summary, DYRK1B localises to PBs, associates with multiple PB components, including DCP1A, EDC3, EDC4, PAT1B, XRN1 and DDX6, and promotes phosphorylation of DCP1A, EDC3, EDC4, PAT1B and 4E-T. Moreover, DYRK1B kinase activity promotes PB abundance (Fig. 6F).

## DISCUSSION

To identify DYRK1B-inducible phosphoproteins, including DYRK1B substrates, we employed an unbiased phospho-SILAC screen. This analysis identified targets in pathways previously linked to DYRK1B, including cell cycle regulation (MPLKIP, RBL1) and chromatin and transcriptional control (MDC1, KDM2A, SET, ARID4A) (Chen et al., 2013; Dong et al., 2021, 2020; Ewton et al., 2003), while also expanding the DYRK1B phosphoproteome to include proteins involved in translational regulation (RPS6, EIF4G2), autophagy (ATG9A, SQSTM1), transporter function (SLC4A7, SLC6A15, SLC7A2) and cytoskeletal organisation (afadin, ABLIM1, CDC42EP1, KATNA1). The detection of previously validated substrates (Dong et al., 2020) supports the robustness of our study. A novel finding from this analysis was the enrichment of proteins associated with mRNA processing and RNP complexes, particularly core components of PBs, including DCP1A, DCP1B, PAT1B, EDC3 and 4E-T. Further experiments confirmed this connection, demonstrating that DYRK1B activity promotes phosphorylation of these PB-associated proteins in cells and is required to maintain their basal phosphorylation (Fig. 2). *In vitro* kinase assays further established DCP1A and 4E-T as direct DYRK1B substrates, with DCP1A undergoing multisite phosphorylation at proline-directed motifs (Fig. 3). Together, these findings identify PB components as a previously unrecognised class of DYRK1B substrates.

We had anticipated that a phosphorylation-defective DCP1A mutant might exert an interfering effect when expressed in cells, perhaps by competing with wild-type DCP1A to access DCP2 or other regulators. Although mutation of multiple DYRK1B-dependent phosphorylation sites in DCP1A substantially reduced phosphorylation, expression of these mutants did not produce an observable phenotype under the conditions tested. One possible explanation is functional redundancy with DCP1B, which shares significant sequence similarity with DCP1A and contains many of the identified phosphorylation sites. DCP1B also scored in our phospho-SILAC screen and co-localised with DYRK1B in pancreatic cancer cells (Fig. 4E,F). It is therefore plausible that compensation by DCP1B limits the impact of DCP1A phosphomutants, and that combined perturbation of both proteins is required to reveal functional consequences.

A central finding of this study is that DYRK1B kinase activity promotes and maintains PB abundance. Several DYRK1B substrates localised to PBs, DYRK1B co-immunoprecipitated with multiple PB components (Fig. 4, Fig. S3) and our data support an association of DYRK1B with these structures (Fig. 4). PBs are dynamic RNP assemblies formed through multivalent interactions, with core factors such as EDC4, DDX6, 4E-T (Standart and Weil, 2018) and LSM14A acting as nucleators (Franks and Lykke-Andersen, 2008; Jonas and Izaurralde, 2013; Parker and Sheth, 2007; Brandmann et al., 2018). It is therefore notable that several PB nucleators or core PB-associated proteins, including EDC4, DDX6 and 4E-T, are DYRK1B substrates or DYRK1B-interacting proteins. As disruption of these interaction networks can impair PB assembly (Brandmann et al., 2018), DYRK1B-dependent phosphorylation of PB proteins could provide a mechanism for modulating PB formation or stability.

Genetic depletion, knockout and rescue experiments demonstrated that DYRK1B kinase activity is required to maintain PB abundance in cells (Figs 5 and 6). To our knowledge, these findings provide the first direct evidence that DYRK1B regulates PB abundance. DYRK1A knockout did not reduce PB abundance under the conditions tested, although increased DYRK1B expression in DYRK1A knockout cells might mask a contribution from DYRK1A. Consistent with this possibility, recent phosphoproteomic analysis of DYRK1A-overexpressing RPE1 cells identified increased phosphorylation of several PB-associated proteins, suggesting that DYRK1A also influences PBs in some contexts (Li et al., 2024). However, whether DYRK1A directly regulates PB abundance or PB homeostasis remains to be determined.

The assembly of membraneless organelles such as PBs is driven by multivalent interactions among protein domains, intrinsically disordered regions (IDRs) and RNA (Banani et al., 2017, 2016), and is highly sensitive to post-translational modifications (Bah and Forman-Kay, 2016; Hofweber and Dormann, 2018). Phosphorylation can modulate condensate behaviour in a context-dependent manner, either promoting or inhibiting assembly. For example, multisite phosphorylation of FUS inhibits condensation (Monahan et al., 2017), whereas phosphorylation of FMRP (FMR1) promotes condensate formation (Tsang et al., 2019). EDC3 phosphorylation has also been linked to PB regulation in cancer cells (Bearss et al., 2021). Interestingly, phosphorylation of DCP1A by JNK or ERK1/2 has been associated with PB dissolution (Chiang et al., 2013; Rzeczkowski et al., 2011; Yu et al., 2024). In this context, DYRK1B-dependent phosphorylation of multiple PB components, including DCP1A, 4E-T and PAT1B, might influence their interaction properties and contribute to PB assembly or stability, although the precise mechanism remains to be defined.

Accumulating evidence now supports a wider role for DYRK family kinases as regulators of biomolecular condensate homeostasis (Álvarez et al., 2003; Gallo et al., 2023; Yu et al., 2019). For example, DYRK1A has been shown to localise to and regulate nuclear speckles (Álvarez et al., 2003), and DYRK3 regulates stress granule dynamics and might act more broadly as a ‘dissolvase’ of membraneless compartments (Wippich et al., 2013). Taken together with our findings, these studies suggest that DYRKs function as conserved signalling regulators of condensate assembly through multisite phosphorylation of IDR-rich proteins.

Our results identify regulation of PB abundance as a previously unrecognised aspect of DYRK1B biology. Although PBs were initially characterised as sites of mRNA decay, they are now understood as dynamic hubs involved in translational control and stress responses (Anderson et al., 2015; Bearss et al., 2021; Hardy et al., 2017). DYRK1B is amplified in a subset of pancreatic cancer and its expression correlates with poor prognosis (Kuuselo et al., 2007). Inhibition of DYRK1B has been proposed as a therapeutic strategy to target quiescent tumour cell populations (Deng et al., 2009) and modulate immune evasion (Brichkina et al., 2024). By regulating the phosphorylation of core PB components, DYRK1B might influence the stability of these condensates and thereby contribute to the adaptive capacity of cancer cells under stress. In this context, phosphorylation of DCP1A and 4E-T might also provide a useful readout or biomarker of DYRK1B activity in cells. In summary, we identify DYRK1B as a regulator of PB homeostasis and establish PB components as a novel class of DYRK1B substrates. These findings extend the DYRK1B interactome and suggest a mechanistic link between DYRK1B activity and post-transcriptional regulation of gene expression in cancer.

## MATERIALS AND METHODS

### Cell lines and culture conditions

HEK293 [American Type Culture Collection (ATCC), CRL-1573], PANC-1 (ATCC, CRL-1469), HTetR, HD1B and HD1B(KD) cells (described in Ashford et al., 2014), including the normal growth medium used for the tetracycline-inducible HEK293 derivatives, have been described previously. Briefly, HTetR cells were generated by transfecting HEK293 cells with pcDNA6/TR (Invitrogen) to stably express the Tet repressor. HTetR cells were subsequently transfected with pcDNA4/TO-FLAG-DYRK1B to generate clones with tetracycline-inducible expression of FLAG-DYRK1 (Ashford et al., 2014). DYRK1B expression in HD1B or HD1B(KD) cells was induced by addition of tetracycline (Sigma-Aldrich, T7660) to a final concentration of 1 μg ml^−1^.

HeLa cells (CCL-2) were obtained from ATCC and maintained in Dulbecco's modified Eagle's medium (DMEM; Thermo Scientific, 41966-029) supplemented with penicillin (100 U ml^−1^), streptomycin (100 μg ml^−1^), L-glutamine (2 mM) and fetal bovine serum (FBS; Gibco, A5256701; 10% v v^−1^). HeLa Flp-In T-REx cells conditionally expressing EGFP–DYRK1B (Terje Johansen laboratory, UiT The Arctic University of Norway, Tromsø, Norway) were maintained in DMEM supplemented with penicillin (100 U ml^−1^), streptomycin (100 μg ml^−1^), FBS (10% v v^−1^) and blasticidin S (Thermo Fisher Scientific, A1113903; 7.5 μg ml^−1^). The generation of these cells was as follows: DYRK1B cDNA was cloned from pDONR221-DYRK1B (obtained from Harvard plasmids repository; https://dnasu.org/DNASU/GetCloneDetail.do?cloneid=41497) into the pDest-Flp-In-EGFP vector (Alemu et al., 2012; generated in the Terje Johansen laboratory) using the Gateway recombination system (Flp-In™ T-REx™, Thermo Fisher Scientific, K650001). pDest-FlpIn-EGFP-DYRK1B was then co-transfected with recombinase expression plasmid pOG44 (Thermo Fisher Scientific, V600520) into the HeLa Flp-In T-REx cells. After 48 h, cells with the gene of interest integrated into the FRT site were selected with 200 μg ml^−1^ of hygromycin B (Sigma-Aldrich, 400051) and 7.5 μg ml^−1^ blasticidin S. Hygromycin-resistant cells were then expanded in the selection medium and later tested for expression by immunoblotting and immunofluorescence. EGFP–DYRK1B expression was induced with tetracycline (1 μg ml^−1^) for the indicated times.

PANC-1 and PaTu 8988T (Leibniz Institute DSMZ, ACC162) pancreatic cancer cells were handled as described previously (Brichkina et al., 2024). Where indicated for imaging experiments in pancreatic cancer cells, the medium was replaced after 24 h with DMEM containing 0.5% FBS and 1% penicillin/streptomycin, and cells were then left untreated or treated for 24 h with vehicle (0.1% DMSO), AZ191 (AstraZeneca; 1 μM) or LCTB21 (Perha Pharma; 1 μM).

All cell lines were routinely passaged before reaching 80% confluence, maintained at 37°C in a humidified atmosphere containing 5% CO_2_, and confirmed to be mycoplasma negative before experiments.

### Generation of knockout and rescue cell lines

CRISPR-mediated depletion of DYRK family kinases in PaTu 8988T cells was performed as described previously (Brichkina et al., 2024). The guide sequences used were 5′-ATATTGTCATGTTACAGAGG-3′ for human *DYRK1A* and 5′-GGTTGTCGTCATCATAACCA-3′ for human *DYRK1B*.

For rescue experiments, DYRK1B-knockout PaTu 8988T cells were transfected with EF-DYRK1B or EF-DYRK1B Y271F/Y273F (Singh et al., 2019) using Helix-In transfection reagent (Oz Biosciences) for 48 h, and then selected with blasticidin S until single clones were obtained.

### Plasmids, mutagenesis, transfection and siRNAs

pEGFP-C1-DCP1A was kindly provided by Michael Kracht (Rudolf Buchheim Institute of Pharmacology, Justus Liebig University Giessen, Giessen, Germany) and was subcloned into pcDNA3.1(+) (Invitrogen, V790-20) to generate pcDNA3.1(+)-HA-DCP1A. DCP1A phosphosite mutants were generated by site-directed mutagenesis to produce the S315A and S319A double mutant, the 9A mutant (S60A, S62A, S142A, S353A, T401A, S522A, S523A, S525A, T531A) and the 11A mutant (S60A, S62A, S142A, S315A, S319A, S353A, T401A, S522A, S523A, S525A, T531A).

Full-length EGFP–DYRK1B, FLAG–DYRK1B and FLAG–DYRK1B-D239A constructs have been described previously (Ashford et al., 2014). pEGFP-C3-DYRK1A was kindly provided by Walter Becker (Institute of Pharmacology and Toxicology, Aachen University, Aachen, Germany), and DYRK2- and DYRK3-coding sequences from the Mammalian Gene Collection were subcloned into pEGFP-C3 (Clontech, 6082-1). HeLa Flp-In T-REx EGFP–DYRK1B kinase-dead derivatives were generated from the parental EGFP–DYRK1B line by Q5 site-directed mutagenesis (New England Biolabs). pcDNA-6Myc-4E-T was kindly provided by Dr Philippe Roux (IRIC, Université de Montréal, Montréal, Canada).

For transient DNA transfection, cells at approximately 70% confluence were transfected with jetPRIME (Polyplus) according to the manufacturer's instructions. In a 12-well format, 0.5 μg DNA was mixed with 75 μl jetPRIME buffer and 2 μl reagent, incubated for 10 min at room temperature, added to cells for 6 h, and then replaced with fresh complete medium for a further 24–48 h.

For knockdown experiments, GFP siRNA (Eurofins), a negative control siRNA (Qiagen, 1022076), ON-TARGETplus human DYRK1B siRNA (Dharmacon, L-004806-00-0005) and Silencer siDYRK1B (Thermo Fisher Scientific, AM51331) were used at a final concentration of 30 nM. siRNAs were transfected using Lipofectamine RNAiMAX (Thermo Fisher Scientific, 13778075) according to the manufacturer's instructions.

### SILAC phosphoproteomics

HD1B cells were grown in SILAC DMEM (Dundee Cell Products) supplemented with dialysed FBS (10%, 10 kDa cut-off), L-proline (84 mg l^−1^), L-glutamine (2 mM), penicillin, streptomycin, blasticidin S and zeocin, and containing either light ^12^C_6_-arginine and ^12^C_6_-lysine (R0K0) or heavy ^13^C_6_-arginine and ^13^C_6_-lysine (R6K6). Cells were maintained for at least ten doublings, which gave 98–99% heavy amino acid incorporation. DYRK1B expression was induced in the heavy-labelled cells by tetracycline (1 μg ml^−1^) for 8 h.

Cells were washed briefly in PBS and lysed in ice-cold modified TG lysis buffer (20 mM Tris-HCl, pH 7.5, 137 mM NaCl, 1 mM EGTA, 10 mM EDTA, 1% Triton X-100, 10% glycerol, 1.5 mM MgCl_2_, 1 mM sodium orthovanadate, 1 mM PMSF, 10 μg ml^−1^ leupeptin, 10 μg ml^−1^ aprotinin and 50 mM NaF). After clarification (12,000 ***g***, 10 min, 4°C), equal amounts of protein from control light-labelled and DYRK1B-induced heavy-labelled lysates were combined.

Aliquots of the lysates containing 100 μg of protein were precipitated with acetone (4 volumes) for 1 h at −20°C. The precipitated proteins were solubilised in 25 mM ammonium bicarbonate, 6 M guanidine hydrochloride and 10 mM dithiothreitol (100 μl) at 50°C for 1 h, then cooled to room temperature and alkylated with iodoacetamide for 30 min in the dark. The *S*-carbamidomethylated proteins were again precipitated with ice-cold acetone then solubilised in 25 mM ammonium bicarbonate and 4 M guanidine hydrochloride containing Lys-C protease (1 μg). After 1 h, the samples were diluted 10-fold with 25 mM ammonium bicarbonate containing trypsin (2 μg) and incubated for 16 h at 50°C. The digestion was terminated by the addition of 10% aqueous trifluoroacetic acid to a final concentration of 0.5%. Phosphopeptides were extracted from the digests with titanium dioxide beads (GL Sciences), eluted from the beads with 5% ammonium hydroxide, then dried and resuspended in 0.1% trifluoroacetic acid.

Phosphopeptides were analysed by LC-MS/MS on a LTQ-Orbitrap Velos mass spectrometer (Thermo Fisher Scientific) coupled to a Proxeon nanoLC system (Proxeon Biosystems, Odense, Denmark). Phosphopeptides were separated on a reversed-phase column (0.05×500 mm ReproSil C18-AQ, 3 μm; Dr. Maisch GmbH, Ammerbuch, Germany) at a flow rate of 80 nl min^−1^ with a gradient of 0–40% acetonitrile (containing 0.1% formic acid) in 8 h. The mass spectrometer scan cycle comprised a high resolution (30,000 at *m*/*z* 400) survey scan, followed by up to 20 MS/MS scans (in higher-energy collisional dissociation mode at 7500 *m/z* 400 resolution), with 120 s dynamic exclusion of former target ions.

The mass spectral data were searched against the human entries in the UniProt database using Mascot software (accessed 2019) and the search results processed using Proteome Discoverer to extract SILAC ratios. Differentially regulated phosphopeptides were subsequently used for motif analysis and for downstream enrichment and interaction analyses shown in Fig. 1. Site-level phosphorylation assignments for relevant phosphopeptides are reported in Table S1 together with localisation probabilities, sequence windows and MS/MS evidence identifiers where available.

### Immunoblotting, immunoprecipitation and Phos-tag analysis

For standard immunoblotting, cells were lysed in ice-cold TG lysis buffer, clarified by centrifugation and quantified before addition of Laemmli sample buffer. In PaTu 8988T experiments, samples were lysed directly in 1× Laemmli buffer. Proteins were resolved by SDS-PAGE, transferred to Immobilon P/PVDF membranes, blocked in TBS containing 0.1% Tween 20 and 5% milk, and probed with the indicated primary and HRP-conjugated secondary antibodies. Signals were visualised by enhanced chemiluminescence. Where indicated, 50 μM Phos-tag was included in the resolving gel.

The primary antibodies used were: anti-DCP1A (Novus Biologicals, H00055802-M06, 1:1000), anti-DDX6 (Bethyl Laboratories, A300-A61A, 1:1000), anti-EDC4 (Cell Signaling Technology, 2548, 1:1000), anti-HA (Santa Cruz Biotechnology, sc-7392, 1:1000), anti-FLAG M2 (Cell Signaling Technology, 14793, 1:1000), anti-V5 (Proteintech, 14440-1-AP, 1:5000), anti-DCP1B D2P9W (Cell Signaling Technology, 13233, 1:1000), anti-p62 (BD Biosciences, 610833, 1:1000), anti-phospho-p62 T269/S272 (Cell Signaling Technology, 13121, 1:1000), anti-XRN1 (Bethyl Laboratories, A300-443A, 1:5000), anti-PAT1B (Bethyl Laboratories, A303-482A, 1:2000), anti-β-actin (Sigma-Aldrich, A5441, 1:10,000), anti-EDC3 (Bethyl Laboratories, A303-986A, 1:2000), anti-GAPDH (Abcam, ab181602, 1:10,000), anti-DYRK1B D40D1 (Cell Signaling Technology, 5672, 1:1000), anti-DYRK1A (Cell Signaling Technology, 2771, 1:1000), anti-c-Myc (Santa Cruz Biotechnology, sc-40; 1:1000), anti-p27KIP1 (Calbiochem, NA35, 1:1000), anti-pS10-p27KIP1 (Santa Cruz Biotechnology, sc-12939-R, 1:500), anti-CCND1 (Calbiochem, CC12, 1:1000), anti-pT286-CCND1 (Cell Signaling Technology, 2921, 1:1000), anti-p21CIP1 (BD Biosciences, 556431, 1:1000), anti-ERK1 (BD Biosciences, 610031, 1:4000), anti-4E-T (Abcam, ab55881, 1:1000), anti-GFP (Roche, 11814460001, 1:1000), anti-DCAF7 (Abcam, ab138490, 1:1000), anti-HSP90 (BD Transduction Laboratories, 610419, 1:1000), anti-phospho-Ser/Thr-Pro (Upstate, 05-368, 1:1000) and anti-phospho-Thr-Pro (Cell Signaling Technology, 9391, 1:5000).

For HA–DCP1A pulldowns, HD1B cells expressing HA-tagged wild-type or mutant DCP1A were induced with tetracycline (1 μM) for 24 h to express FLAG–DYRK1B, lysed in TG buffer and incubated with anti-HA agarose (Thermo Fisher Scientific, 26181) for 3 h at 4°C. For endogenous EDC4 immunoprecipitation, lysates prepared in immunoprecipitation buffer (50 mM Tris-HCl pH 7.5, 150 mM NaCl, 1 mM EDTA, 0.1% Triton X-100, 10% glycerol, phosphatase and protease inhibitors) were incubated with anti-EDC4 antibody (Cell Signaling Technology, 2548, 1:1000) and TrueBlot anti-rabbit beads (Rockland Immunochemicals, 00-8800-25) for 3 h at 4°C.

For FLAG immunoprecipitations, HEK293 cells were transfected with the indicated FLAG-tagged constructs, lysed after 24 h, and incubated overnight at 4°C with anti-FLAG M2 magnetic beads (Sigma-Aldrich, M8823). Beads were washed three times with TG lysis buffer and bound proteins were eluted in 2× sample buffer.

For GFP-Trap immunoprecipitation, HeLa Flp-In T-REx EGFP–DYRK1B cells were treated with tetracycline for 24 h and, where indicated, consecutively treated with DYRK inhibitors [AZ191 (1 μM), harmine (Sigma-Aldrich, 286044; 1 μM) or GSK626616 (Sigma-Aldrich, SML3355; 1 μM)] for 8 h. For cytosolic fractions in Fig. 4C, cells were washed in ice-cold PBS, scraped into 500 μl of ice-cold isotonic fractionation buffer (20 mM HEPES, pH 7.2, 250 mM sucrose, 0.5 mM EDTA, 0.5 mM Na_3_VO_4_, 20 μM leupeptin, and 10 μg ml^−1^ aprotinin) and processed using a modified version of a previously published protocol (Putcha et al., 2001). Clarified cytosolic lysates were incubated with GFP-Trap magnetic beads (ChromoTek, gtma) for 2 h at 4°C and washed three times before elution in sample buffer.

For V5 immunoprecipitation from PaTu 8988T cells stably expressing V5-tagged DYRK1B, cells were lysed in PBS containing 1% Triton X-100 and protease inhibitors. After centrifugation (10,000 ***g***, 10 min, 4°C), lysates were pre-cleared with rabbit IgG and Protein A magnetic beads (Invitrogen, 10334693) and then incubated for 4 h at 4°C with anti-V5 antibody (Proteintech, 14440-1-AP, 1:5000) or control rabbit IgG together with magnetic beads. Beads were washed five times in lysis buffer and boiled in 1× Laemmli buffer. See Fig. S4 for all uncropped immunoblots.

### *In vitro* kinase assays and targeted phosphosite mapping

For *in vitro* kinase assays, HEK293 cells were transfected with pEGFP-C1-DCP1A constructs and EGFP–DCP1A proteins were isolated 24 h later by immunoprecipitation. Bead-bound EGFP–DCP1A was incubated with recombinant GST–DYRK1B (Invitrogen, PV4649) in kinase buffer containing 50 mM Tris-HCl pH 7.5, 0.1 mM EGTA, 0.1% 2-mercaptoethanol, 10 mM MgCl_2_ and 0.1 mM ATP for 60 min at 30°C, with or without AZ191 (1 μM) or harmine (Sigma-Aldrich, 286044; 1 μM). Reactions were terminated by boiling in Laemmli buffer and analysed by immunoblotting.

For targeted mapping of DCP1A phosphorylation sites, three DCP1A preparations were analysed: endogenous DCP1A immunoprecipitated from HD1B cells after tetracycline treatment (24 h), EGFP–DCP1A transiently expressed in HD1B cells before tetracycline induction, and EGFP–DCP1A isolated from HEK293 cells and phosphorylated *in vitro* by recombinant DYRK1B. Immunocomplexes were resolved by SDS-PAGE, gels were stained with Coomassie Blue, and the DCP1A bands were excised and split into two samples for downstream mass spectrometric analysis, essentially as described previously (Webster and Oxley, 2005). For each sample, one gel portion was digested with trypsin and the other with chymotrypsin. Peptides were analysed by LC-MS/MS on the same system used for the SILAC analysis, but using a 0.075×150 mm column (Dr. Maisch GmbH, Ammerbuch, Germany) with a 30 min gradient of 0–40% acetonitrile with 0.1% formic acid.

### Immunofluorescence, confocal imaging and super-resolution microscopy

For high-content quantification of PBs in HTetR, HD1B, HD1B(KD) and PANC-1 cells, cells were grown on glass coverslips (poly-L-lysine-coated coverslips for HTetR/HD1B derivatives), treated as indicated, washed briefly in PBS and fixed/permeabilised in ice-cold methanol for 7 min. Coverslips were blocked in PBS containing 2% BSA and 0.02% sodium azide and incubated overnight at 4°C with anti-DCP1A (Novus Biologcals, H00055802-M06, 1:200) and anti-DDX6 (Bethyl Laboratories, A300-A61A, 1:1000) antibodies. After washing, coverslips were incubated with fluorescent secondary antibodies [Alexa Fluor 568 anti-mouse-IgG (Invitrogen, 1:1000) and Alexa Fluor 647 anti-rabbit-IgG (Invitrogen, 1:1000)], mounted in VectaShield with DAPI, and imaged at 40× magnification on an InCell 6000 instrument (GE Healthcare, UK). Images were analysed in CellProfiler (Broad Institute). Foci containing DCP1A or DDX6 were identified, and PBs were defined as cytoplasmic foci positive for both markers. At least 150 cells were analysed per sample.

For siRNA-based PB analysis, PANC-1 cells were transfected with control or DYRK1B-targeting siRNAs, fixed 48 h later and processed as above.

For confocal analysis of DYRK family localisation, HeLa cells were transiently transfected with an empty EGFP vector or EGFP–DYRK1A, EGFP–DYRK1B, EGFP–DYRK2 or EGFP–DYRK3 constructs and fixed with 4% paraformaldehyde for 15 min at room temperature. Cells were permeabilised in 0.2% Triton X-100, blocked in 1% BSA, 5% normal goat serum and 0.02% Triton X-100, and incubated with anti-DCP1A (Novus Biologicals, H00055802-M06, 1:200), anti-DDX6 (Bethyl Laboratories, A300-A61A, 1:1000) or anti-HA (Santa Cruz Biotechnology, sc-7392, 1:500) antibodies, followed by incubation with Alexa Fluor-conjugated secondaries [Alexa Fluor 568 anti-mouse (Invitrogen, 1:1000) and Alexa Fluor 647 anti-rabbit (Invitrogen, 1:1000)]. Nuclei were counterstained with DAPI and coverslips were mounted in Dako fluorescence mounting medium. Images were acquired as maximum-intensity projections on a Zeiss LSM780 confocal microscope using a 63× oil-immersion objective. Line-scan co-localisation analyses were performed in Zeiss ZEN and plotted in GraphPad Prism.

For super-resolution imaging of inducible HeLa Flp-In T-REx EGFP–DYRK1B cells, cells were grown on high-precision no. 1.5H coverslips (Marienfeld Superior, Lauda-Königshofen, Germany; 0107032), induced with tetracycline as indicated, fixed and stained for DCP1A and DDX6, mounted in ProLong Diamond Antifade (Invitrogen, P36961) and imaged on an N-SIM S microscope (Nikon) equipped with a 100×, 1.49 NA oil-immersion objective and an Andor iXon 897 camera. Three-dimensional structured illumination microscopy images were reconstructed in NIS-Elements (Nikon) and processed in Fiji.

For imaging of endogenous DYRK1B and DCP1B in PaTu 8988T cells, cells were seeded on glass coverslips. After 24 h, cells were washed with PBS and the medium was changed to starving conditions (DMEM, 0.5% FBS, 1% penicillin/streptomycin). Cells were either left untreated for 24 h or were treated with 0.1% DMSO (vehicle control), 1 μM LCTB21 (kindly provided by Perha Pharma; Lindberg et al., 2023) or 1 μM AZ191 for 24 h prior to fixing. Cells were then fixed in 4% formaldehyde for 10 min at room temperature, permeabilised with 0.5% Triton X-100 for 5 min, blocked in PBS containing 10% FBS, and incubated overnight at 4°C with anti-DYRK1B (Santa Cruz Biotechnology, sc-390417, 1:200) and anti-DCP1B (Cell Signaling Technology, 13233, 1:100) antibodies in PBS containing 10% goat serum and 0.1% saponin, followed by incubation with Alexa Fluor-conjugated secondary antibodies [Alexa Fluor 488 anti-mouse (Invitrogen, 1:400) and Alexa Fluor 633 anti-rabbit (Invitrogen, 1:400)] for 2 h at room temperature. Coverslips were mounted in VectaShield with DAPI.

For deconvolved widefield imaging in PaTu 8988T cells, *z*-stacks were acquired on a Leica DM5500 B widefield microscope and subjected to three-dimensional deconvolution and maximum-intensity projection. Quantification of DCP1B-positive PBs from these images was performed in ImageJ.

Stimulated emission depletion (STED) imaging was performed on a Leica STELLARIS STED microscope (Leica Microsystems, Wetzlar, Germany) using an HC PL APO CS2 93×/1.30 GLYC objective. Excitation was provided by the white light laser and a 405 nm laser as appropriate for the fluorophores used. Images were acquired with a theoretical pixel size of 60 nm, with depletion at 592 nm and 775 nm and photon-counting τSTED (fluorescence lifetime-based STED) detection.

### Live-cell imaging

For live-cell imaging of DYRK1B and PBs, PaTu 8988T cells were transiently co-transfected with DYRK1B–EGFP and mCherry–DDX6 (generated using pT7-EGFP-C1-HsRCK, Addgene #25033, and mCherry2-C1, Addgene #54563) expression constructs using polyethylenimine (Polysciences). The culture medium was replaced after 24 h with fresh DMEM supplemented with 10% FBS and 1% penicillin/streptomycin prior to imaging.

Live-cell imaging was performed on the Leica STELLARIS STED microscope. Confocal images were acquired to monitor the dynamics of DYRK1B–EGFP and mCherry–DDX6. Imaging was carried out using the same fluorescence excitation and detection settings as described for super-resolution microscopy, but without application of STED depletion lasers.

### Quantifications and statistics

High-content imaging data presented in this study were derived from at least three independent biological replicates. Image analysis, graph generation and statistical tests were performed using GraphPad Prism (versions 8–10; GraphPad, San Diego, CA, USA). Details of data analysis are provided in the corresponding figure legends.

Software and online resources used for image analysis and figure preparation were CellProfiler (Broad Institute, https://cellprofiler.org; McQuin et al., 2018), GraphPad Prism (GraphPad, https://graphpad.com), Fiji/ImageJ (National Institutes of Health, https://fiji.sc), Zeiss ZEN (Zeiss, https://zeiss.com), NIS-Elements (Nikon, https://nikon.com) and BioRender (https://www.biorender.com/).

PB quantification from CellProfiler-based assays was analysed using one-way ANOVA with appropriate multiple comparison tests, as indicated in the figure legends. Quantification of DCP1B foci in pancreatic cancer cell imaging experiments, including knockout and rescue conditions, was analysed using unpaired two-tailed *t*-tests. Data are presented as mean±standard deviation (s.d.) or standard error of the mean (s.e.m.), as specified in the figure legends.

Phospho-SILAC data were analysed to generate the Fig. 1D volcano plot. A clean phosphopeptide-only dataset was derived from Table S1 (Source tab) by filtering for phosphopeptides quantified in at least two replicates and excluding reverse hits. The volcano plot displays average log_2_(heavy/light) SILAC ratios against −log_10_(*P*-values), calculated from the peptide level *P*-values. Differentially abundant phosphopeptides were defined as those with a mean fold change (FC) >1.5 and *P*<0.05. The ratio, log_2_FC, mean FC and *P*-value columns used to support Fig. 1D are provided in Table S1. Rows marked ‘Y’ in the ‘Plotted in volcano (Fig. 1D)’ column in Table S1 correspond to phosphopeptides included in the plotted volcano analysis.

STRING (version 12.0, https://string-db.org/) network analysis was performed for the proteins with significantly higher phosphopeptide abundance in the tetracycline-treated versus untreated conditions (interaction confidence >0.4) and STRING was used for the GO annotation of proteins in this network. GO term enrichment was determined by DAVID Bioinformatics (v2023q2 Knowledgebase, Huang et al., 2009; https://davidbioinformatics.nih.gov/) for proteins with peptides exhibiting a mean ≥1.5-fold increase.

## Supplementary Material



10.1242/joces.265054_sup1 Supplementary information

Table S1. DYRK1B SILAC phosphoproteomics data.

Table S2. DCP1A targeted phosphoproteomics data.
