# Supplementary material for: Phosphoproteomics identifies the DYRK1B protein kinase as a regulator of processing bodies
Source: J Cell Sci. 2026 Sep 15;139(17):jcs265054. doi: 10.1242/jcs.265054 (PMC13615683; doi:10.1242/jcs.265054)
Supplement: Supplementary file 1 [file joces-139-265054-s1.pdf]

DCP1A p-Ser^{525}

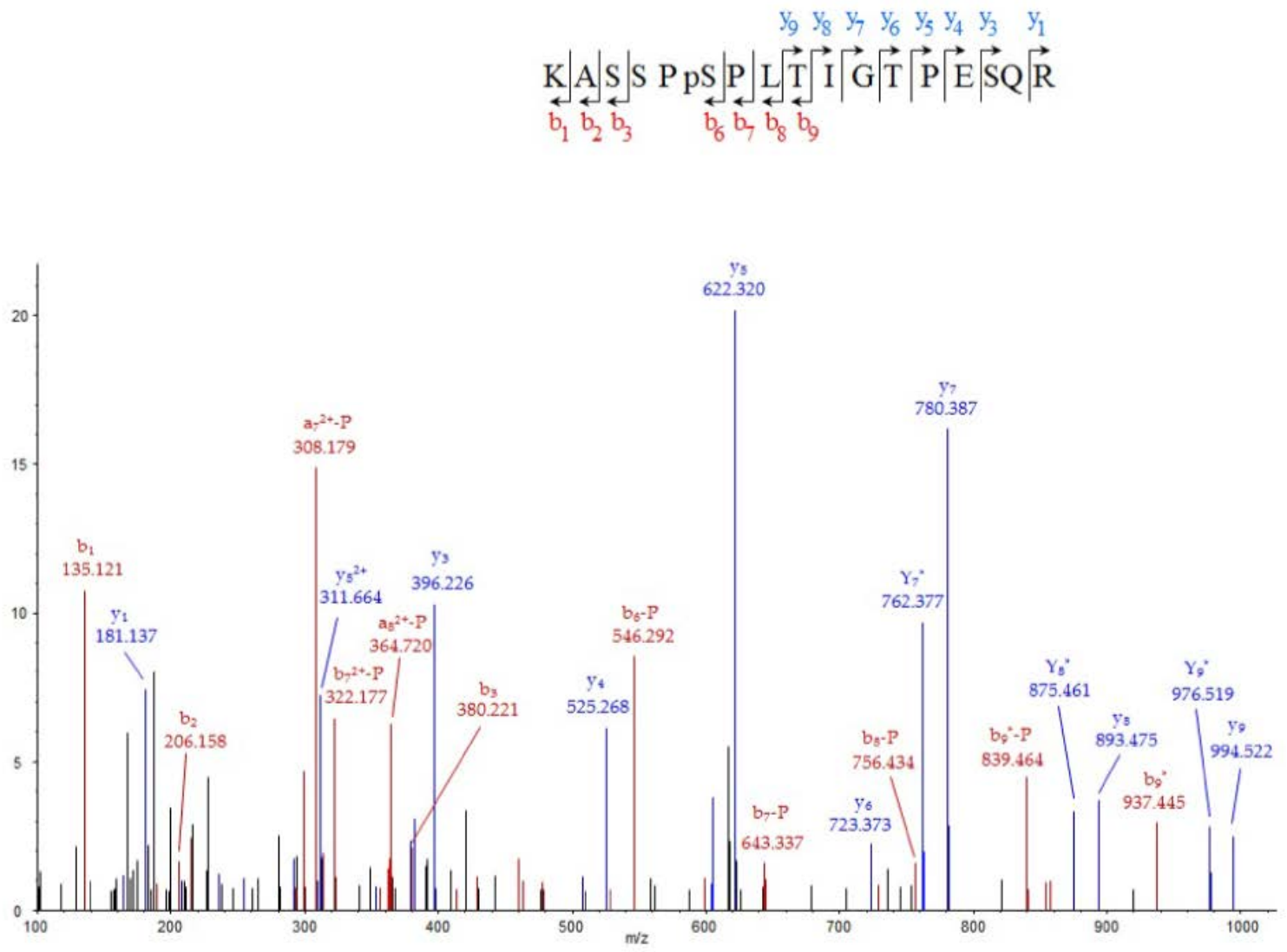

Fig. S1 (related to Fig. 1). Spectrum graph from SILAC mass spectrometry data showing phosphorylated DCP1A peptides.

Journal of Cell Science • Supplementary information

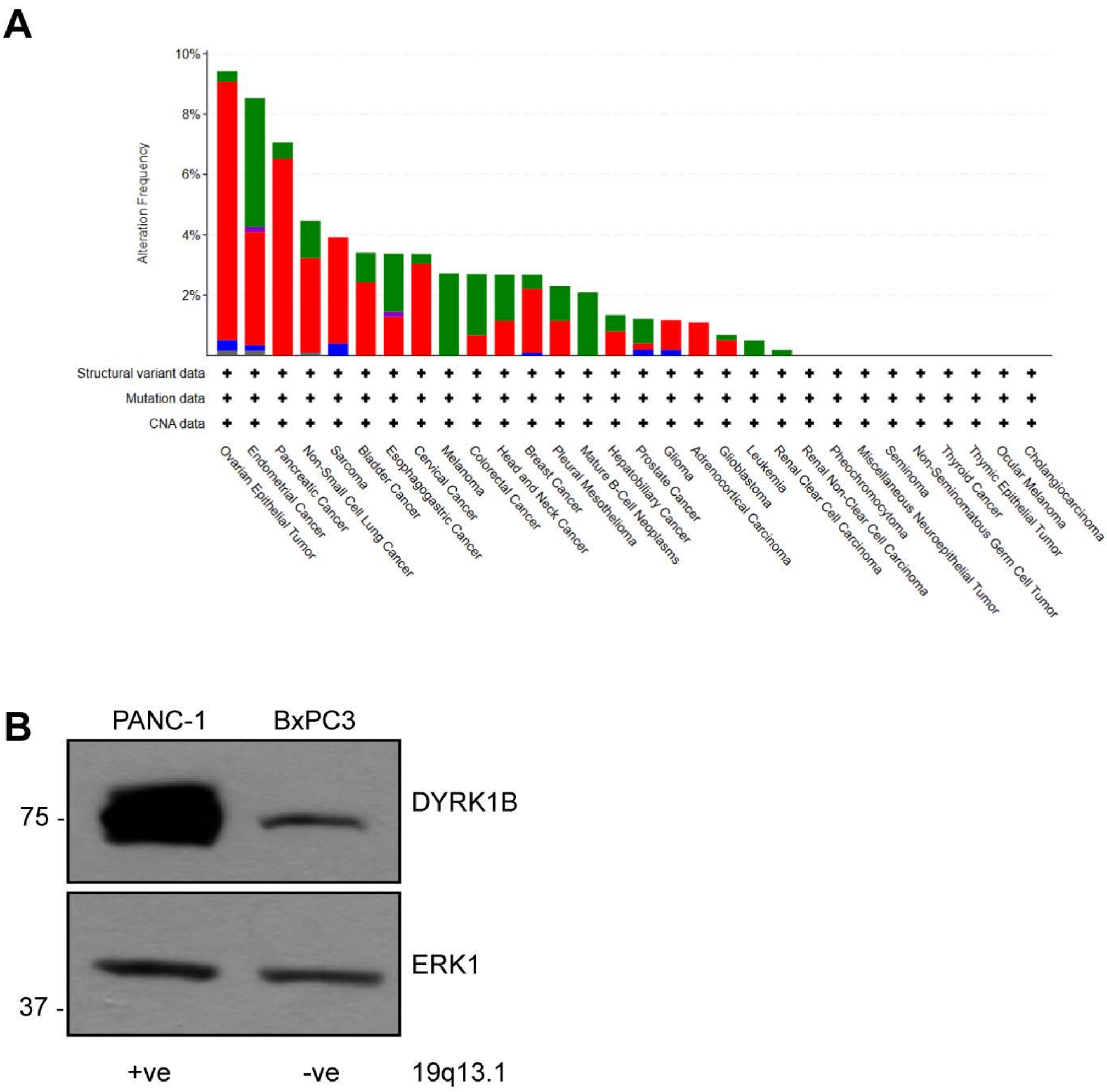

Fig. S2 (related to Fig. 2). DYRK1B amplifications in cancer

(A) Graph obtained from c-bioportal, showing 7% of pancreatic cancer show DYRK1B amplifications. (B) Western blot showing pancreatic cancer cell line (PANC-1) which harbours 19q13.1 amplification exhibiting higher levels of DYRK1B compared to a different pancreatic cancer cell line BxPC3 which does not have 19q13.1 chromosomal amplification.

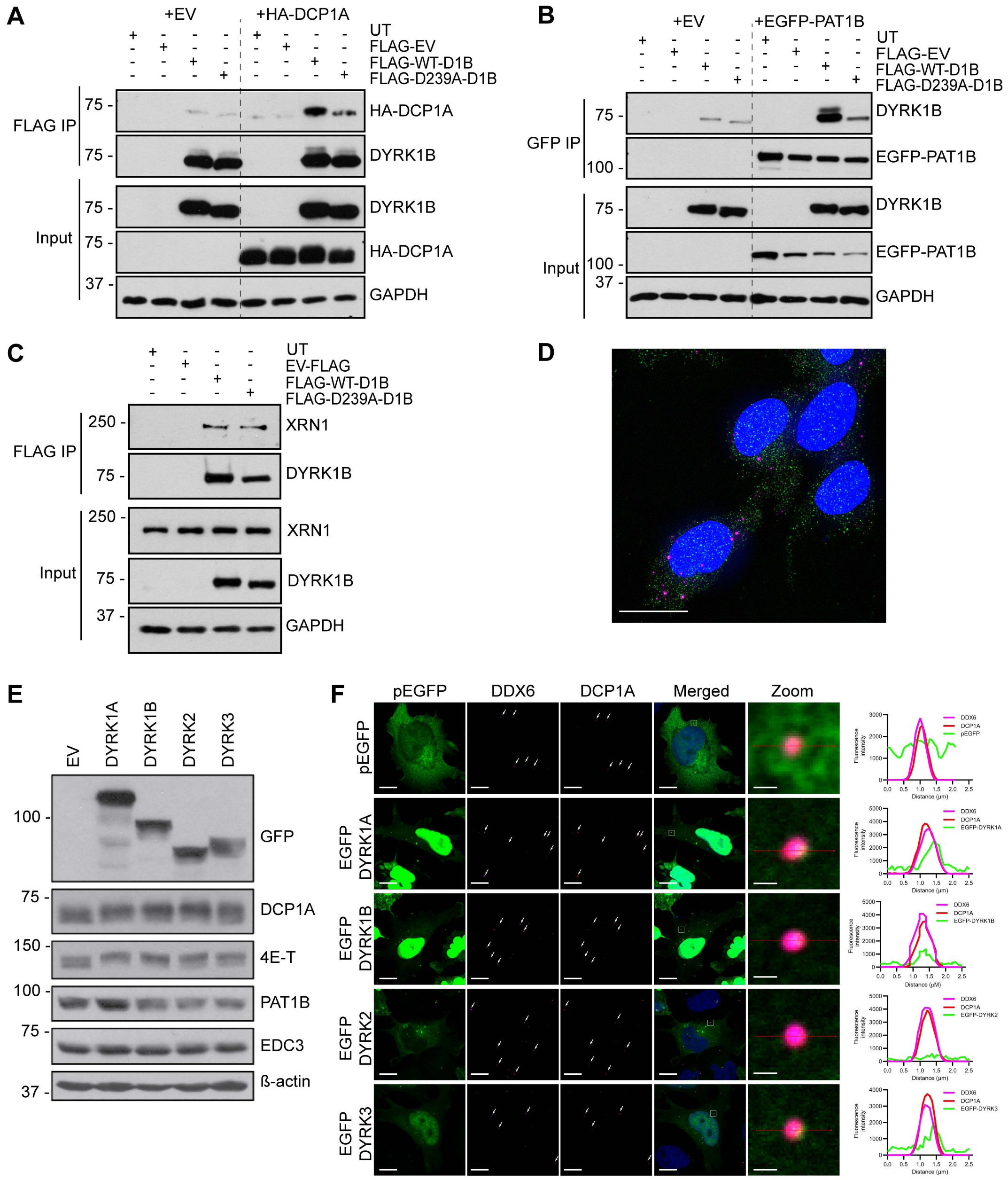

Fig. S3 (related to Fig. 4). P-body components interact with and co-localise with DYRK1B in PBs. (A) FLAG IP of FLAG-WT-D1B or FLAG-D239A-D1B transfected HEK293T cells co-transfected with empty vector (EV) control or HA-DCP1A associates with HA-DCP1A. (B) GFP trap IP of FLAG-WT-D1B or FLAG-D239A-D1B transfected HEK293T cells co-transfected with empty vector (EV) control or EGFP-PAT1B associates with both WT and kinase-dead DYRK1B. (C) FLAG IP of FLAG-WT-D1B or FLAG-D239A-D1B transfected HEK293T cells associates with endogenous XRN1. (D) 3D-deconvoluted widefield image of PaTu 8988T cells stained for endogenous

Journal of Cell Science • Supplementary information

DYRK1B (green) and DCP1B (magenta) showing colocalization. Scale bar in the whole cell image represents 20µm. (E) HEK293 cells transfected with EGFP-tagged DYRK family members. DYRK1A and DYRK1B overexpression produced modest mobility changes in DCP1A and 4E-T, consistent with increased phosphorylation of these PB associated proteins. Effects on PAT1B and EDC3 were less apparent under these conditions. (F) Confocal images of HeLa cells transiently transfected with either control empty vector EGFP or EGFP-tagged DYRK familymembers and stained for DDX6 (magenta) and DCP1A (red). Scale bars, 10µm. Line scan analyses of fluorescence intensities of PBs (DDX6 and DCP1A) along with the various EGFP-tagged DYRK vectors.

Figure 1B

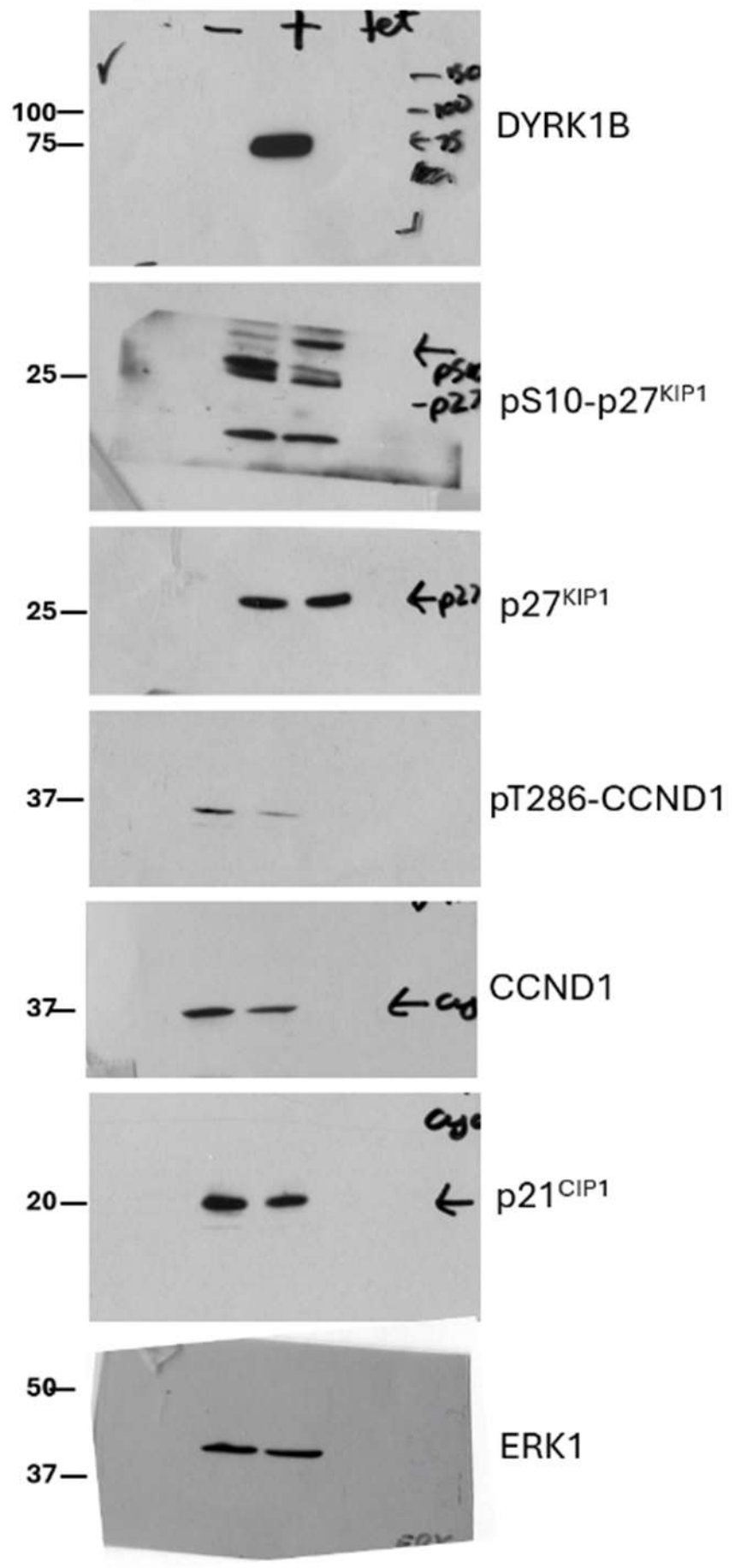

Figure 2A

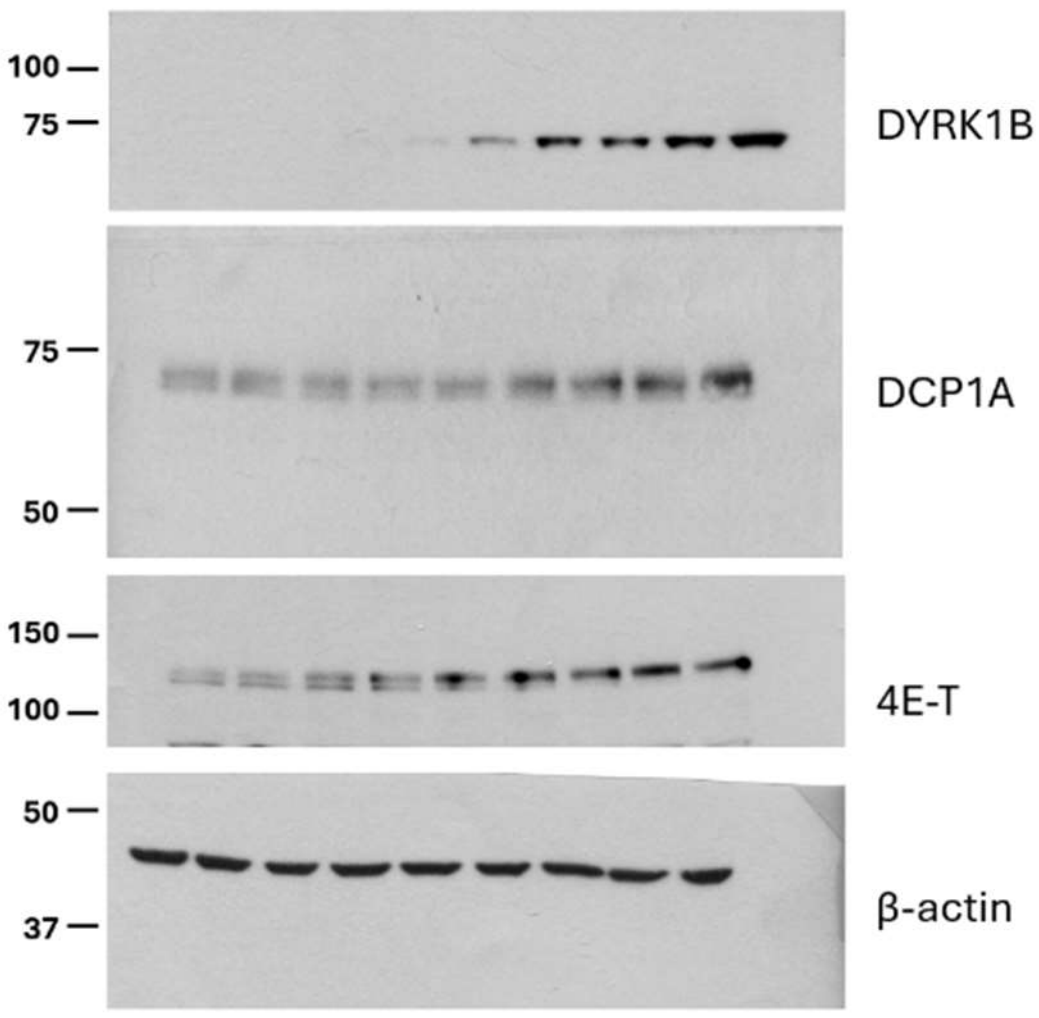

Journal of Cell Science • Supplementary information

Figure 2B

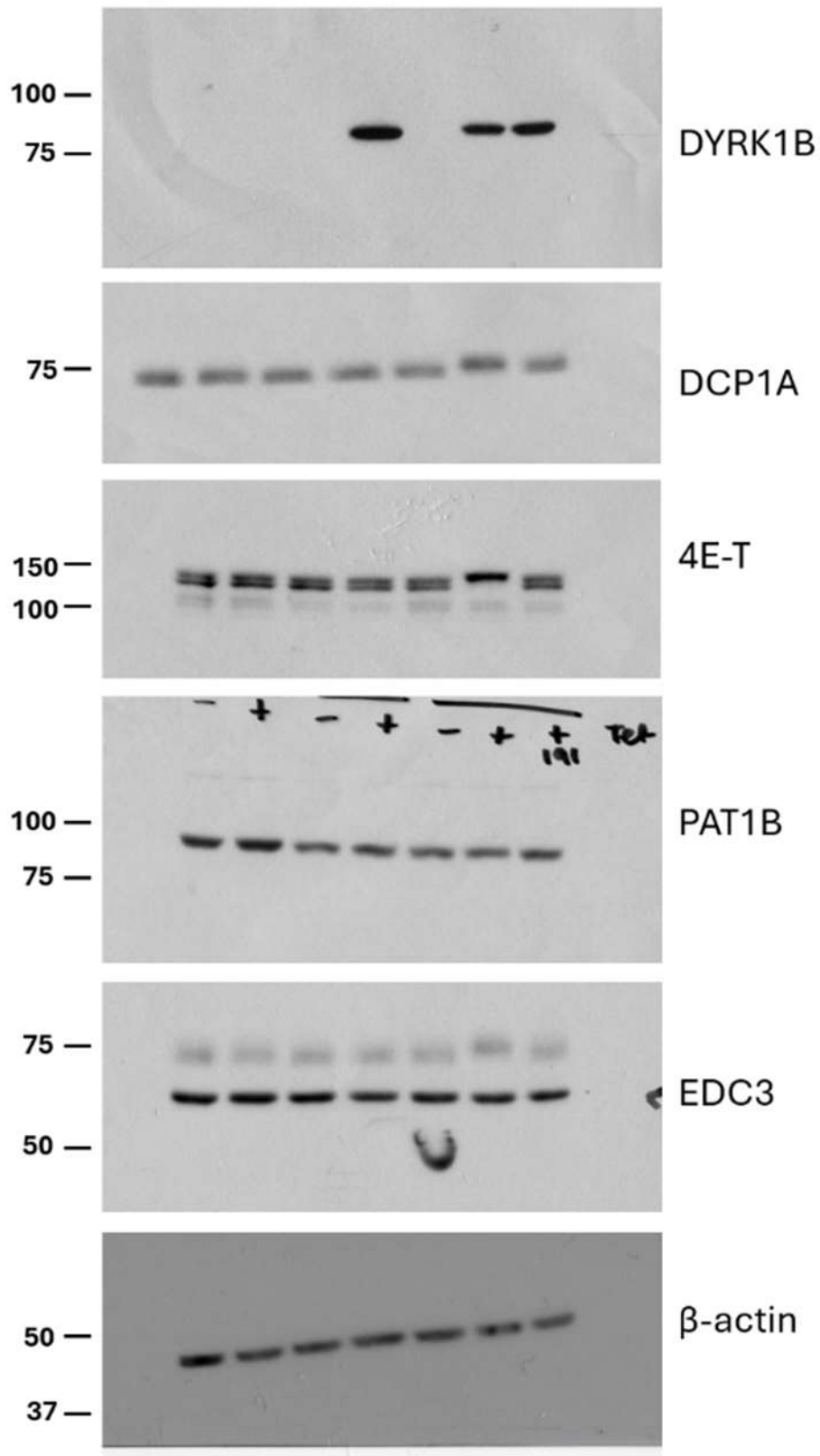

Figure 2C

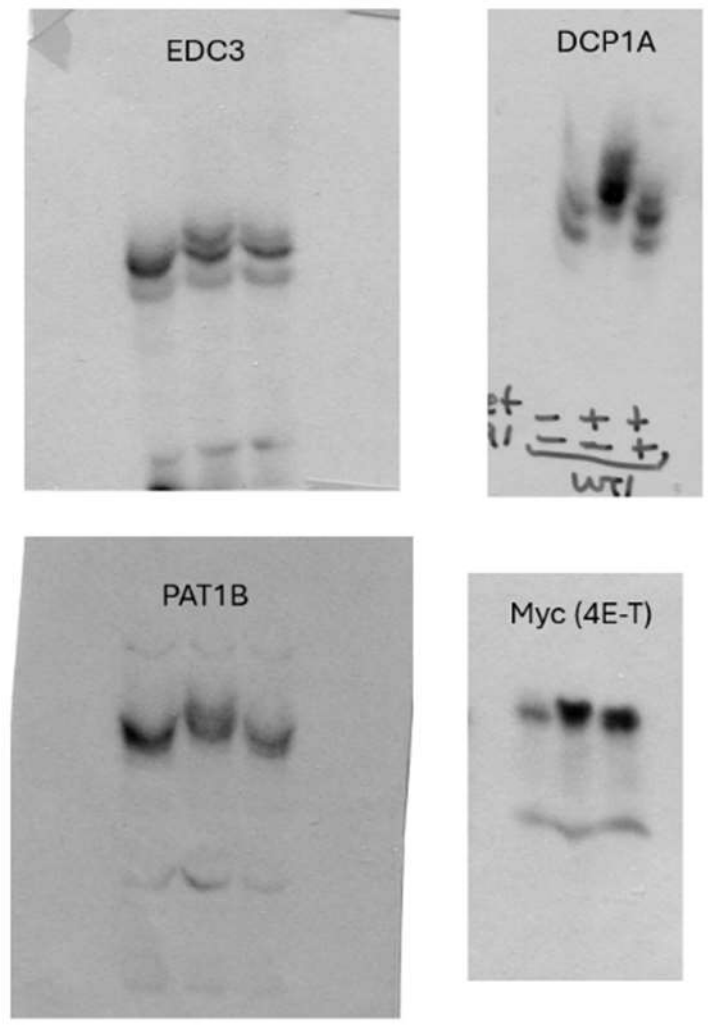

Figure 2D

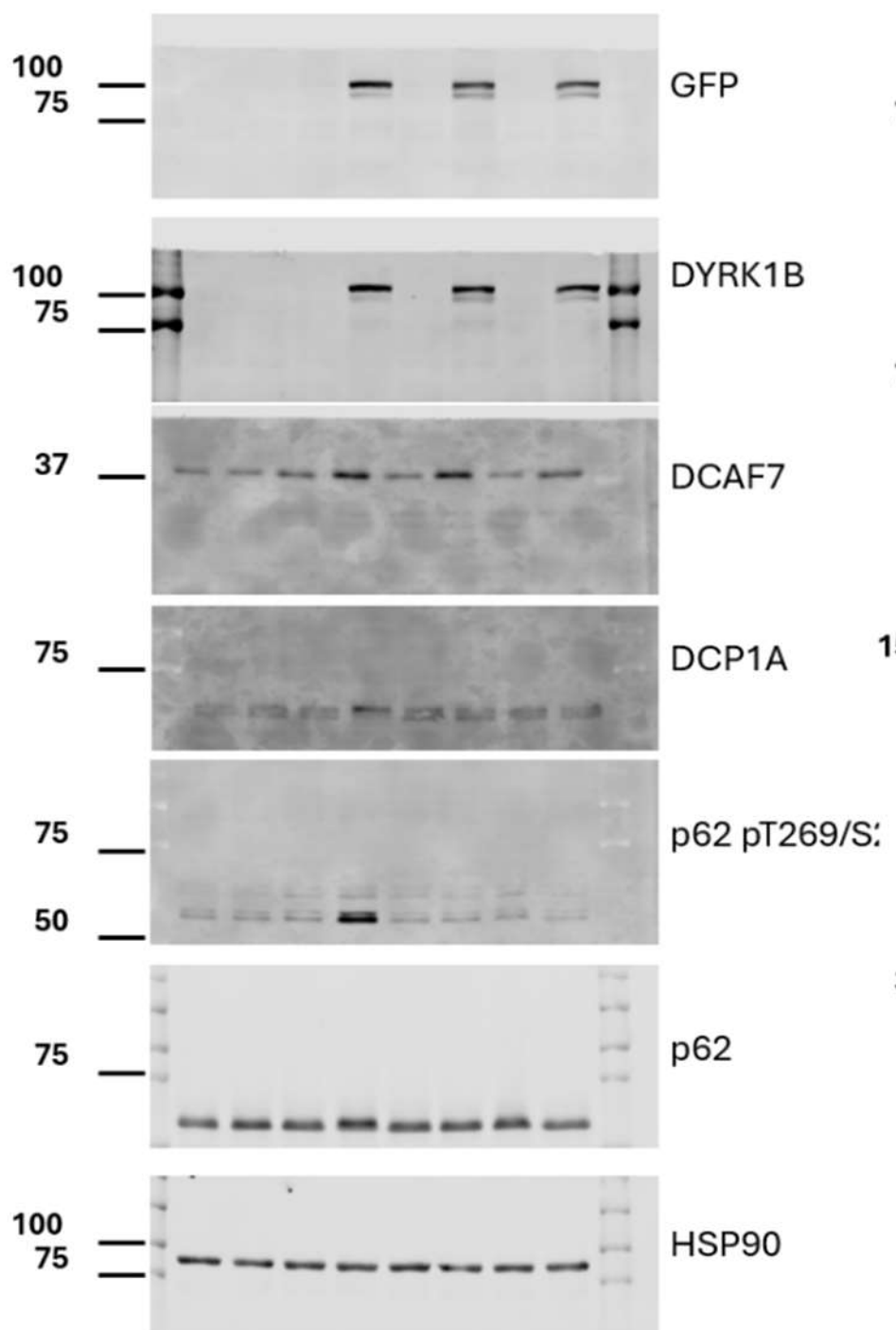

Figure 2E

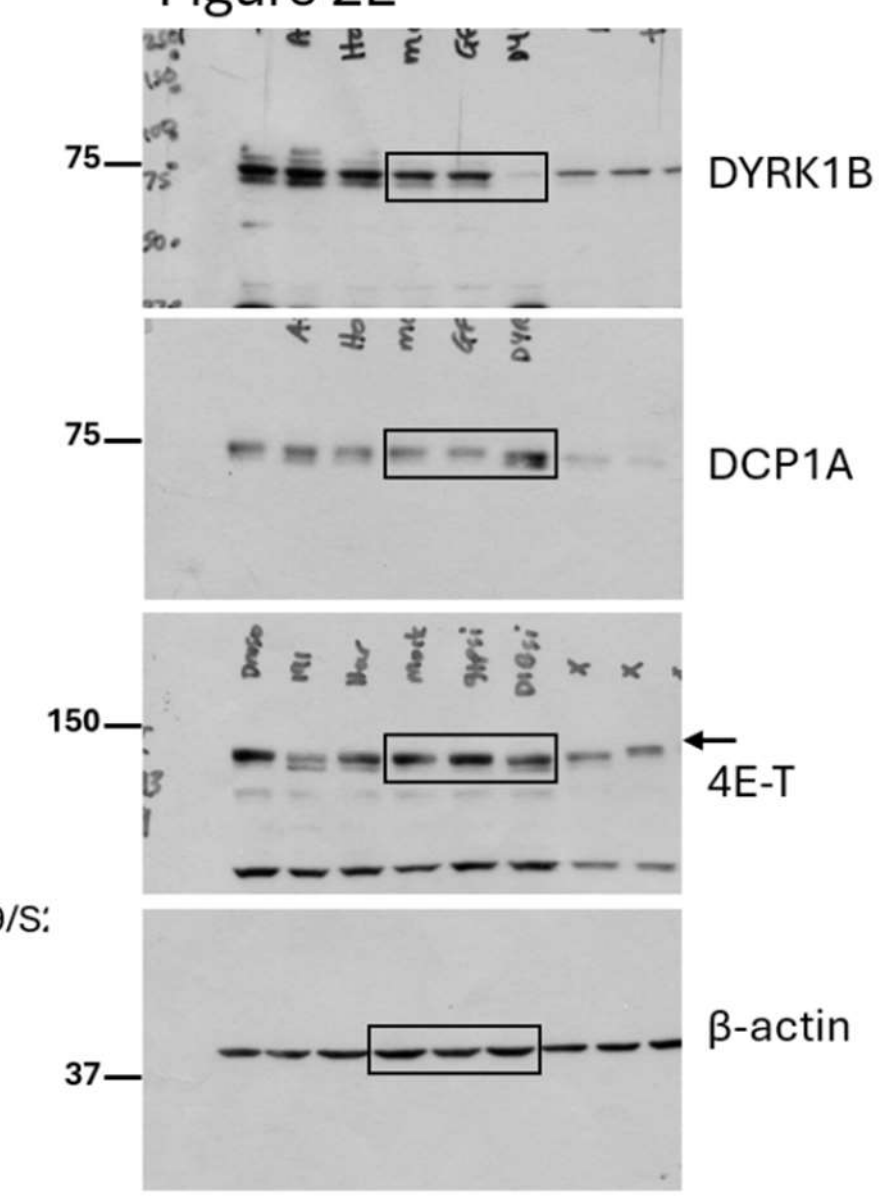

Figure S2B

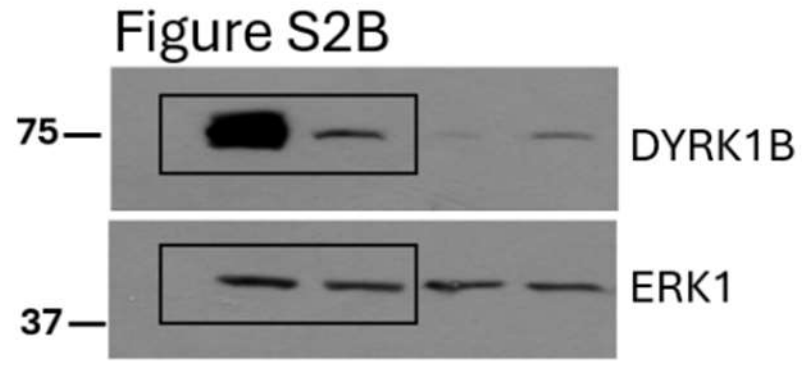

Figure 3A

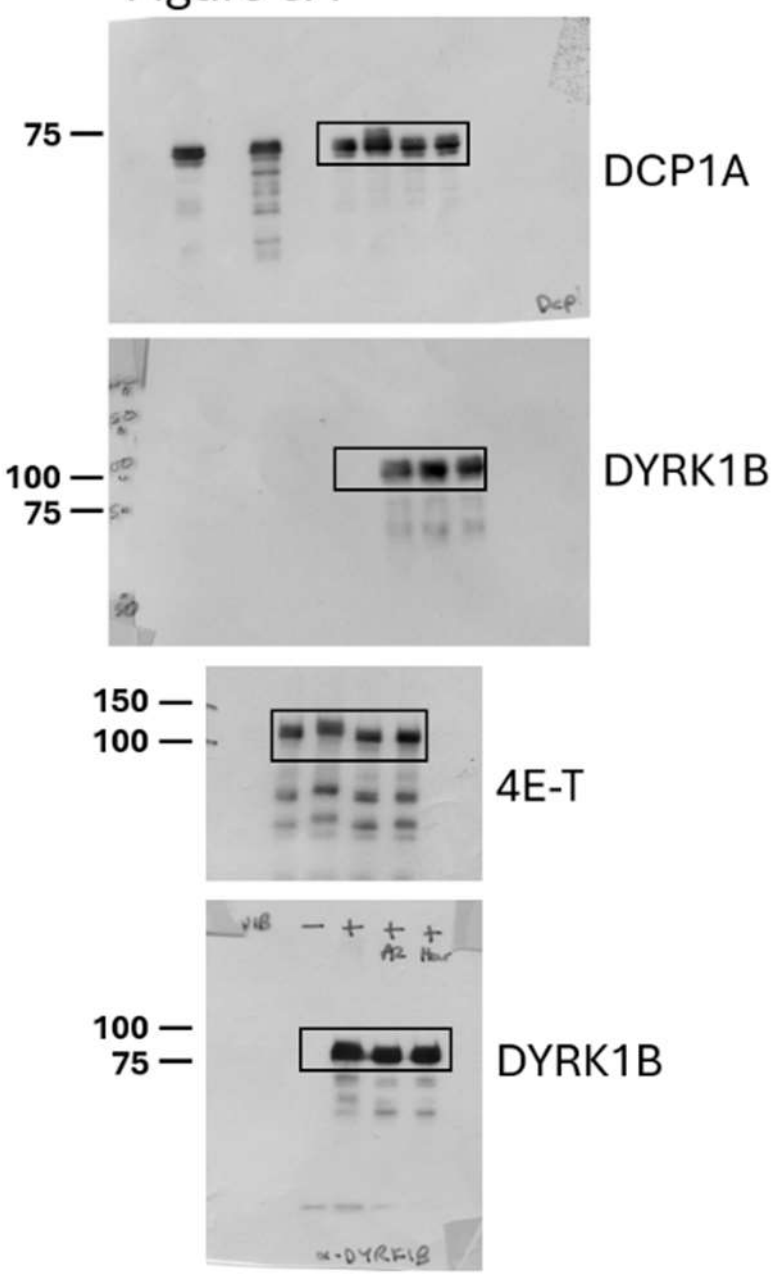

Figure 3B

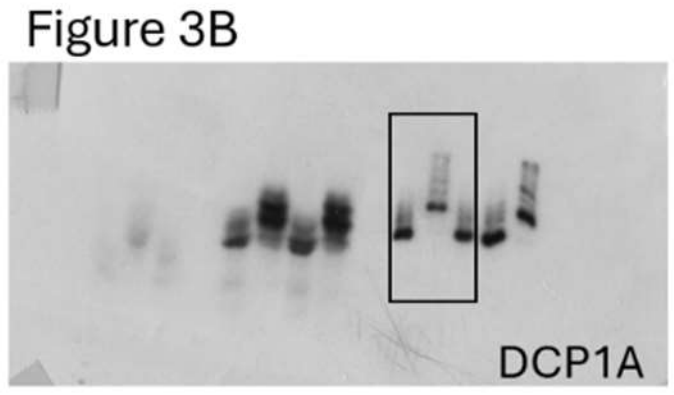

Figure 3D

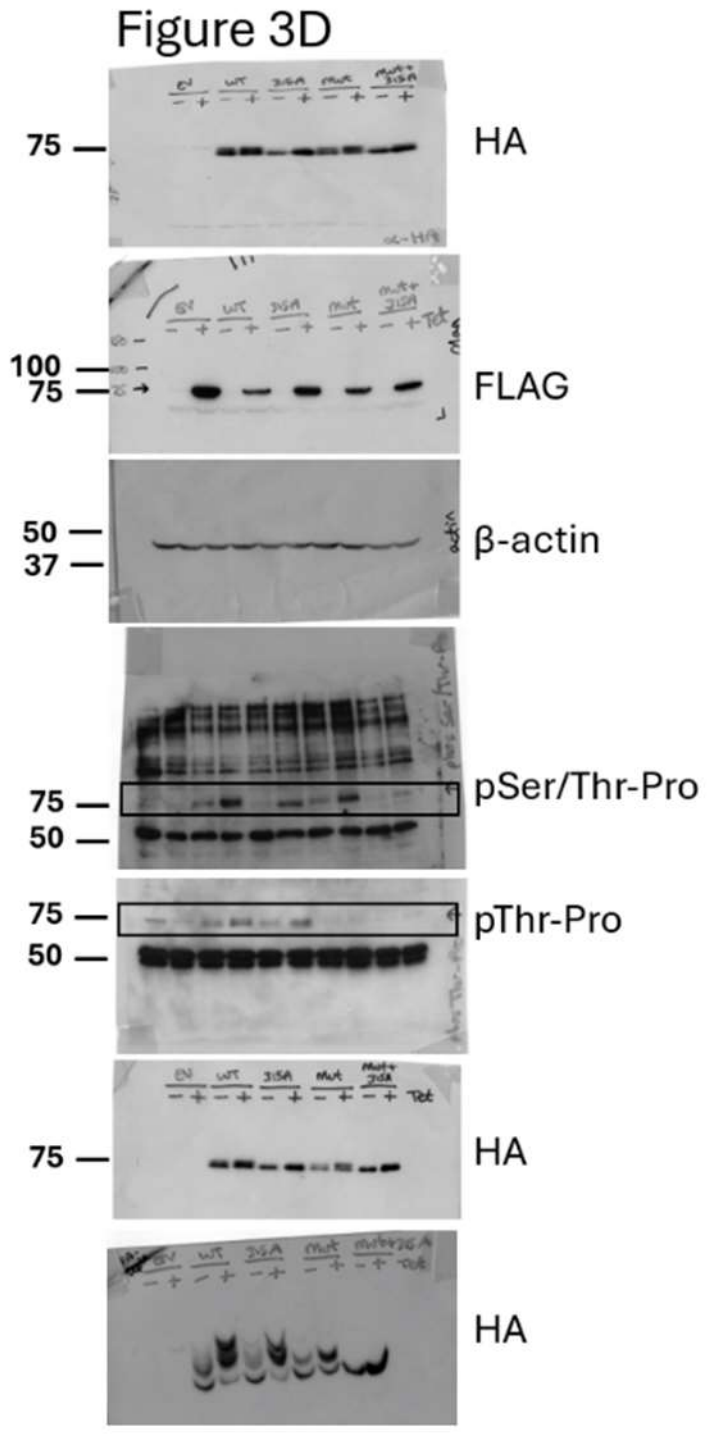

Figure S3A

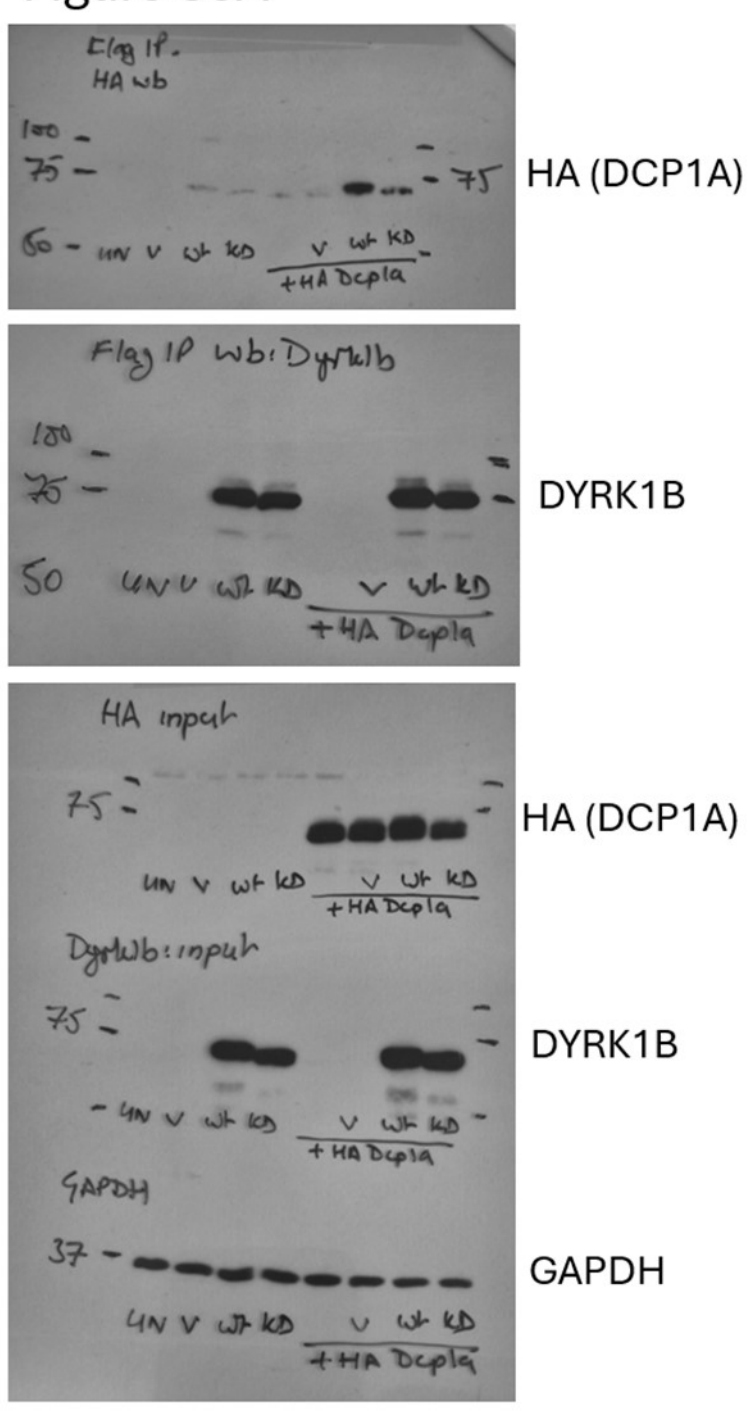

Figure S3B

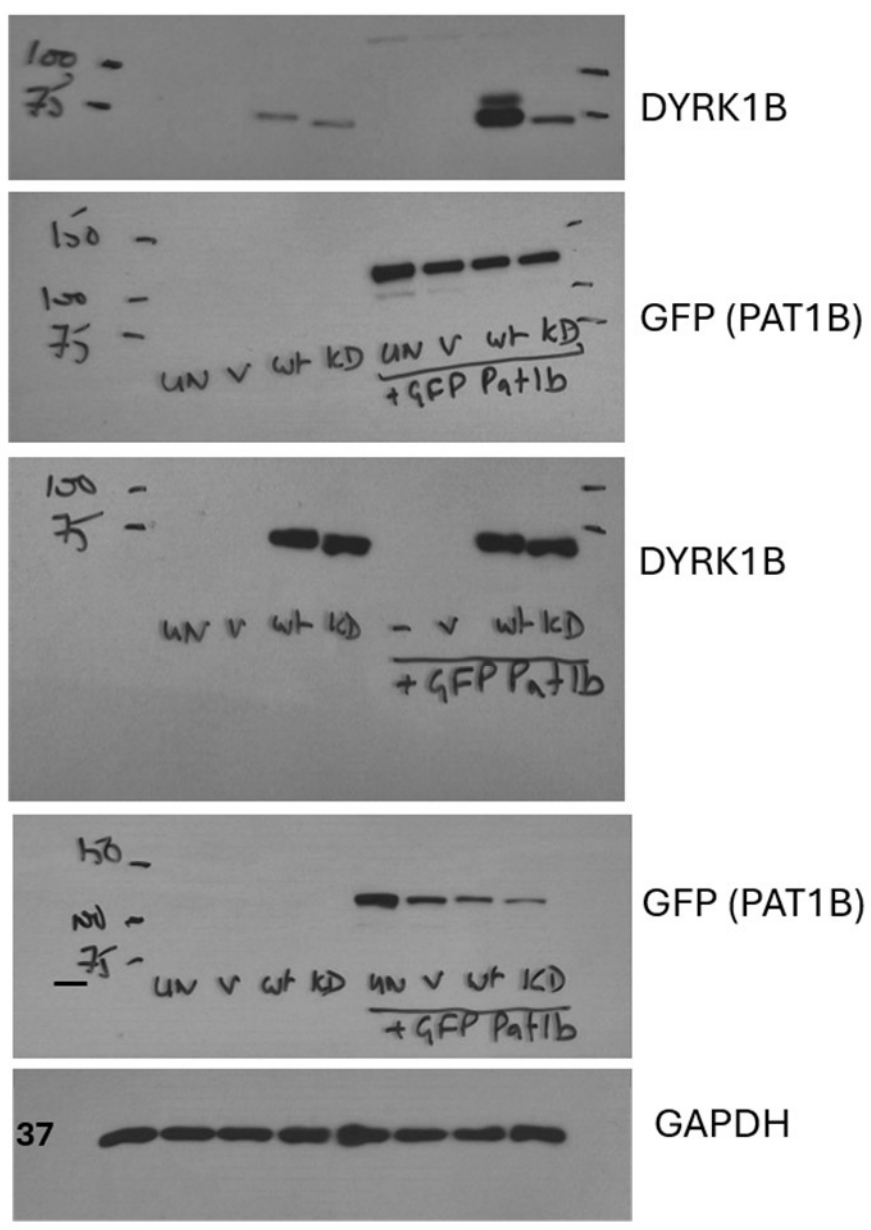

Figure S3C

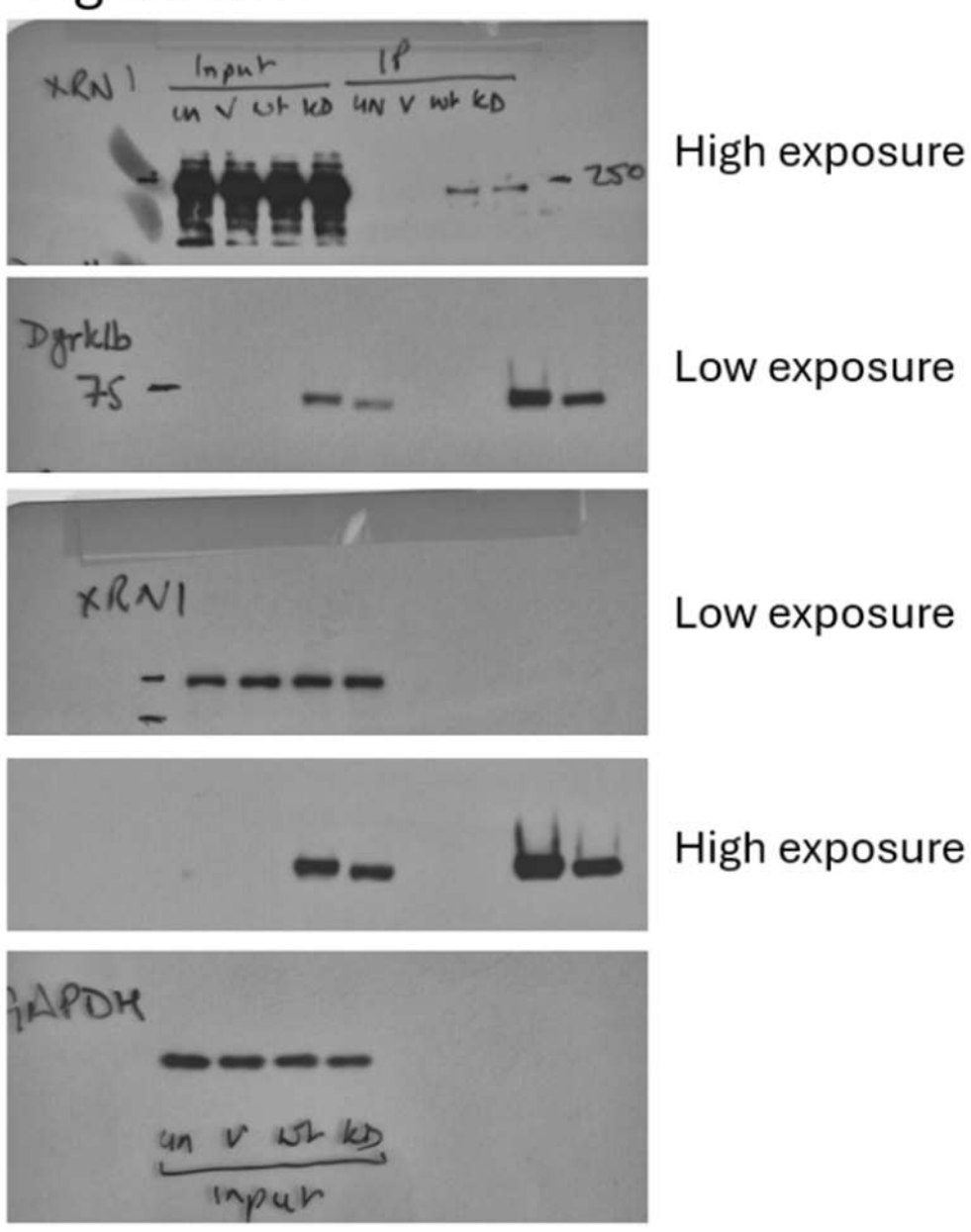

Figure S3E

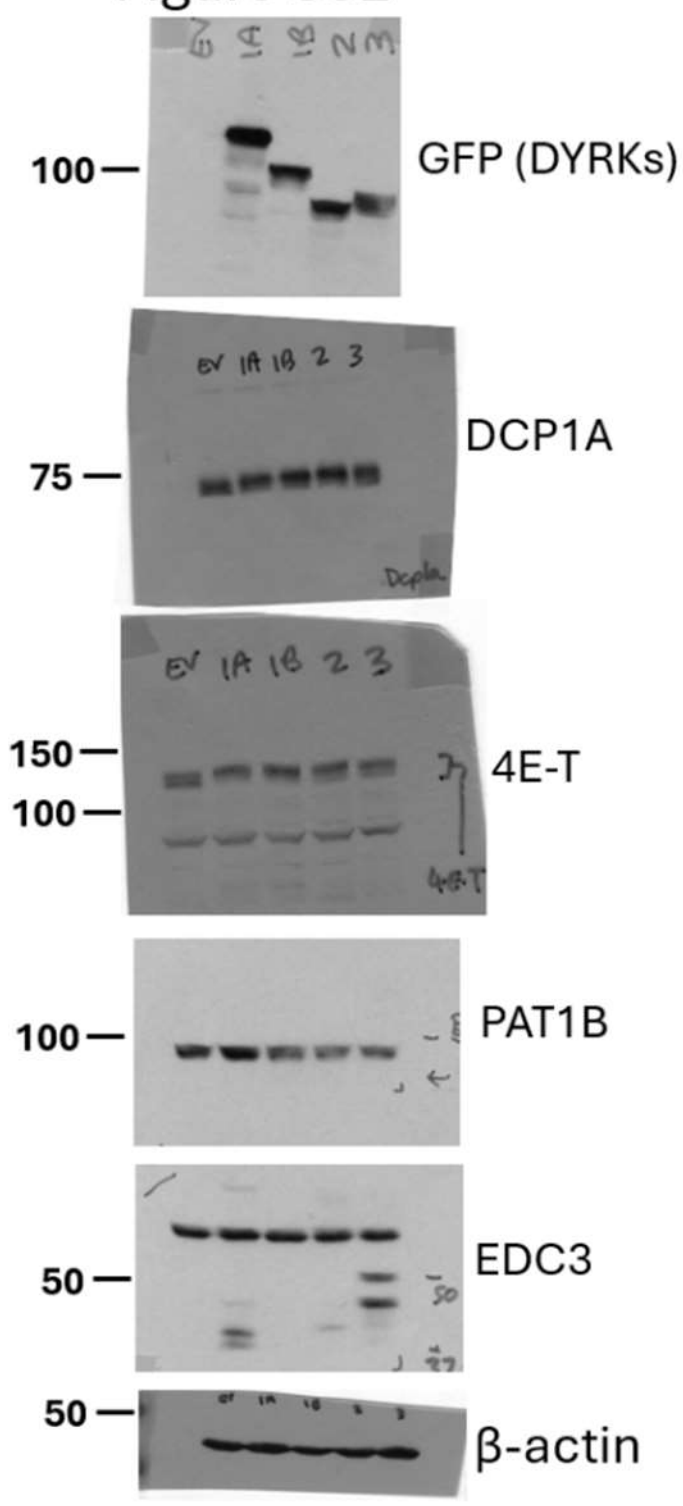

Figure 4A

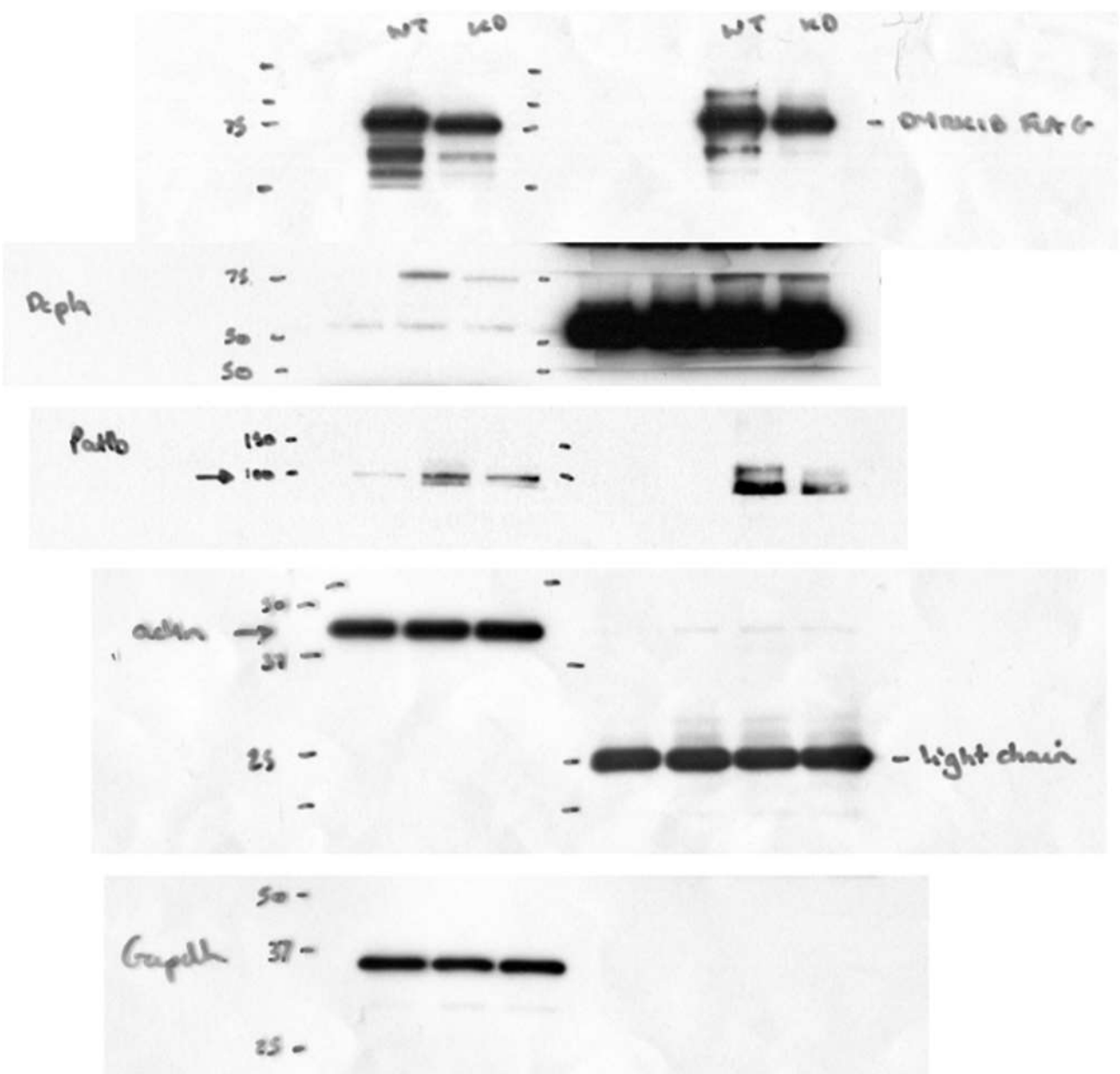

Figure 4B

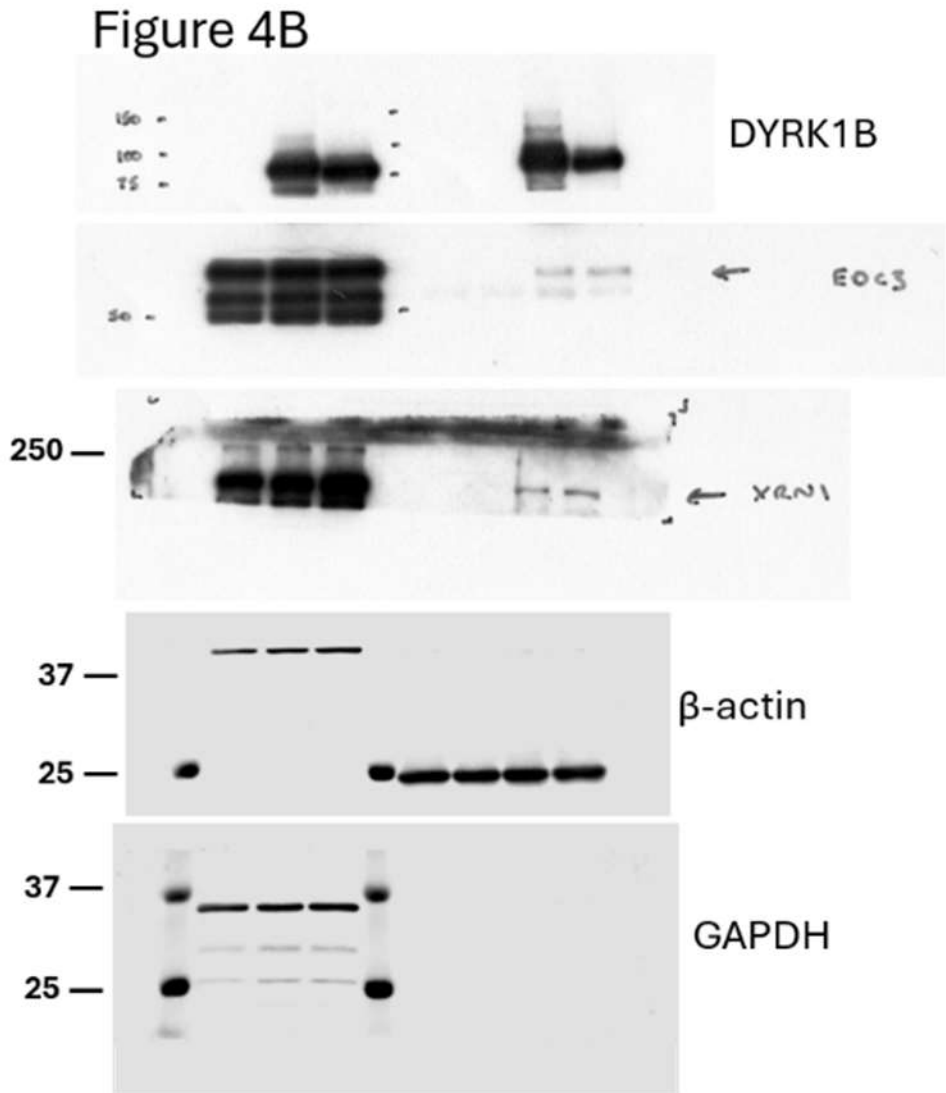

Journal of Cell Science • Supplementary information

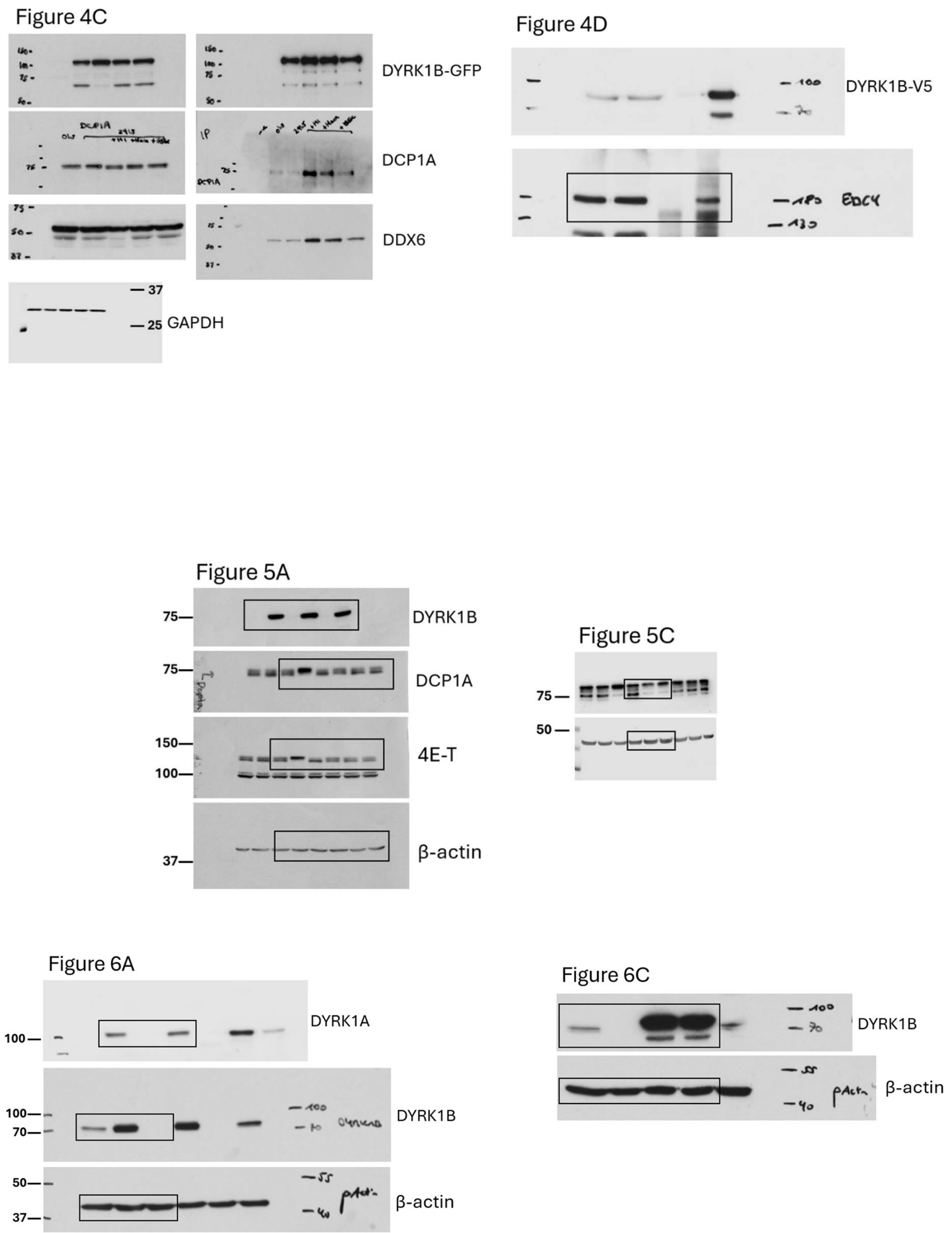

Fig. S4. Uncropped immunoblot results.

Table S1. DYRK1B SILAC phosphoproteomics data.

Available for download at
https://journals.biologists.com/jcs/article-lookup/doi/10.1242/jcs.265054#supplementary-data

Table S2. DCP1A targeted phosphoproteomics data.

Available for download at
https://journals.biologists.com/jcs/article-lookup/doi/10.1242/jcs.265054#supplementary-data

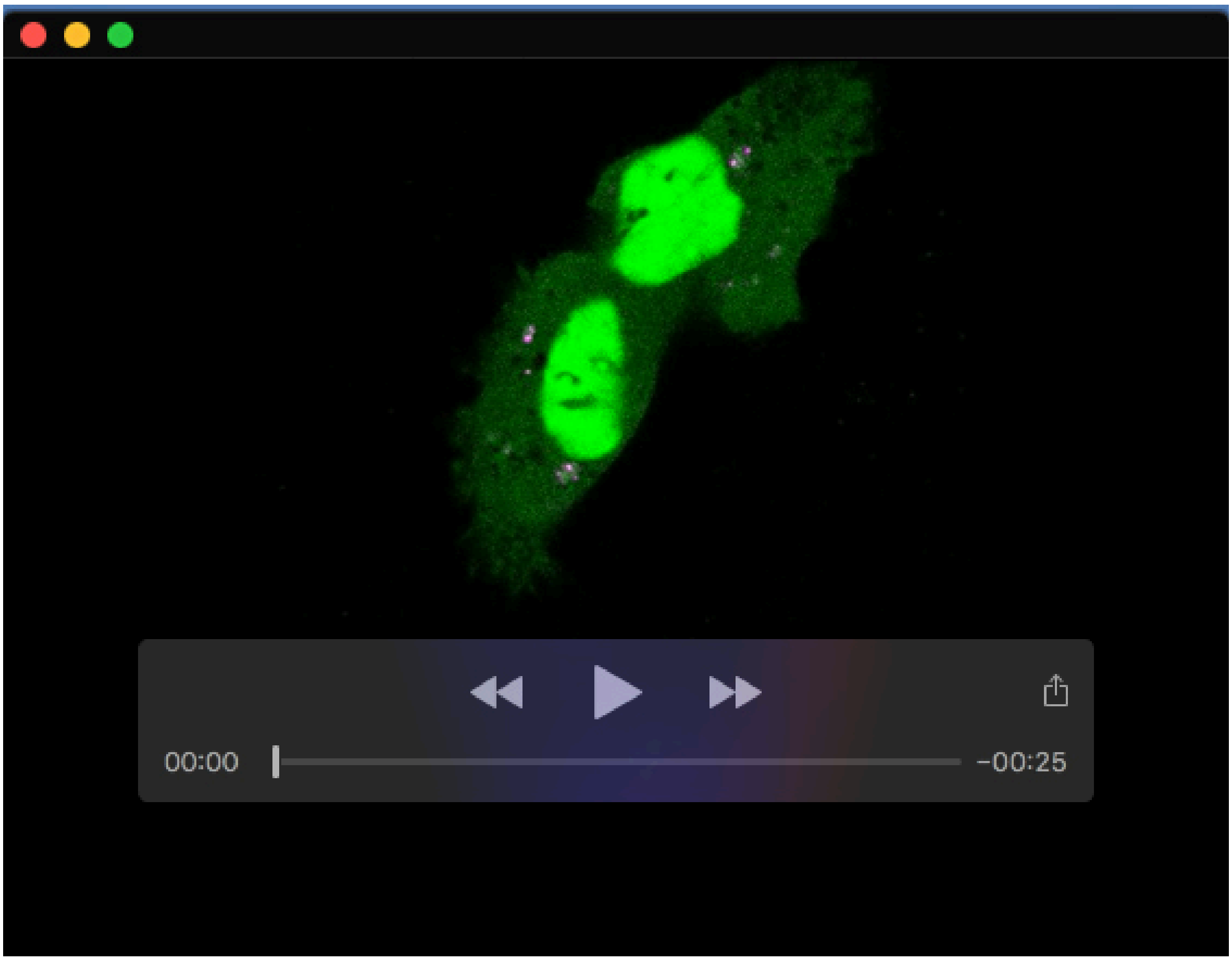

Movie 1. Live-cell confocal imaging of PaTu 8988T cells transiently expressing DYRK1B-GFP and mCherry-DDX6. The movie shows cytoplasmic DYRK1B positive puncta overlapping with DDX6 positive at PBs.
