# Supplementary material for: Phosphoproteomics identifies the DYRK1B protein kinase as a regulator of processing bodies
Source: J Cell Sci. 2026 Sep 15;139(17):jcs265054. doi: 10.1242/jcs.265054 (PMC13615683; doi:10.1242/jcs.265054)
Supplement: Supplementary file 2 [file joces-139-265054_review_history.pdf]

Journal of Cell Science

Phosphoproteomics identifies the DYRK1B protein kinase as a regulator of processing bodies

Anne L. Ashford, Suzan Ber, Miriam S. Ems, Emma Duncan, Kathryn Balmanno, Hannah Reeves, Rachael Huntly, Megan A. Cassidy, Harvey E. Johnston, David Oxley, Thaddeus Mutugi Nthiga, Terje Johansen, Marie Kluge, Ralf Jacob, Matthias Lauth and Simon J. Cook
DOI: 10.1242/jcs.265054

Editor: Caroline Hill
Review timeline
Original submission: 15 May 2026
Editorial decision: 18 June 2026
First revision received: 13 July 2026
Accepted: 31 July 2026

Original submission

First decision letter

MS ID#: jcs.265054

MS TITLE: Phosphoproteomics identifies the DYRK1B protein kinase as a regulator of processing bodies

AUTHORS: Anne L Ashford; Suzan Ber; Miriam S Ems; Emma Duncan; Kathryn Balmanno; Hannah Reeves; Rachael Huntly; Megan A Cassidy; Harvey E Johnston; David Oxley; Thaddeus M Nthiga; Terje Johansen; Marie Kluge; Ralf Jacob; Matthias Lauth; Simon J Cook

ARTICLE TYPE: Research Article

Dear Dr Cook,

We have now reached a decision on the above manuscript.

As you will see, the reviewers gave favourable reports but raised some critical points that will require amendments to your manuscript. I hope that you will be able to carry these out because I would like to be able to accept your paper.

Reviewer 1

SUMMARY OF THE ADVANCE MADE IN THIS PAPER AND ITS POTENTIAL SIGNIFICANCE TO THE FIELD

Ashford et al. have identified key components of processing bodies (PBs) as substrates of the protein kinase DYRK1B. The authors provide convincing evidence that DYRK1B physically associates with and phosphorylates several PB proteins and localizes to PBs. Most importantly, they demonstrate in multiple cell lines that catalytically active DYRK1B is required to maintain normal cellular PB abundance.
Together, these findings establish PB components as a previously unrecognized class of DYRK1B substrates. Given the established roles of DYRK1B in cellular stress responses and cancer cell survival, the identification of DYRK1B as a regulator of PB dynamics may have important implications for stress adaptation mechanisms in cancer cells. Thus, the present study provides

© 2026. Published by The Company of Biologists under the terms of the Creative Commons Attribution License (https://creativecommons.org/licenses/by/4.0/).

valuable new insights both into the cellular functions of DYRK1B and into the emerging field of PB regulation.
Overall, the results are clearly presented and, in general, support the conclusions drawn by the authors. However, I suggest that the authors revise certain aspects of the discussion regarding the proposed role of DYRK1B, as outlined below. Additional specific comments are provided below.

SUGGESTIONS TO AUTHORS

Major comments [Please request additional experiments only if they are essential for supporting the conclusions; authors should be encouraged to highlight any claims that are preliminary or speculative, or to discuss any pitfalls or alternative interpretations in a 'Limitations' section]

Minor comments

Figure 1C (motif analysis) color coding is not obvious and should be explained
Figure 1D: In contrast to the figure legend, significantly altered phosphopeptides are highlighted in black rather than red. I suggest to highlight the P body proteins. The table underlying the volcano plot should be also be provided as supplemental material to make it easier for readers to evaluate the complete dataset.
Table S1: The table should include information about the data presented (metadata, legend to identify the columns) in an extra sheet. This table seems to include both phosphopeptides, unmodified peptides, and those with other modifications. The volcano plot illustrates only phosphopeptides. The table directly underlying the volcano plot could be provided here as an extra sheet (or as an extra file).

Page 6 line 57: "increased phosphorylation of Tyrosine in the activation loop (IYQYIQSR)"
Is this rather the increased abundance of this phosphopeptide rather than increased phosphorylation (meaning a ratio of phosphorylated vs unphosphorylated peptide)?
Page 7, lines 43-47: "many of the DYRK1B-driven phosphorylation sites on DCP1A, DCP1B, 4E-T and PAT1B identified in the screen were proline-directed"
I could not easily track this information in the data (table S1). column 1 lists the identified peptides, but where is the identification of phosphorylation sites by the MS/MS scans? It would helpful the readers to provide a table of the phosphopeptides relevant to this study with the phosphosites indicated, as well as FC and p values (including SQSTM1 pT269, which is also relevant here, and possibly SF3B1 pT434 as a kind of control for the dataset, given that CDKN1B was not detected).
Page 7 line 57: "DYRK1B-selective inhibitor AZ191" the selectivity of AZ191 for DYRK1B over DYRK1A varies in different assays, and at a concentration of 1 μM, DYRK1A is likely also inhibited to some degree. While this is not critical in this experiment with overexpressed DYRK1B, effects on endogenous DYRK1 must be interpreted with caution (see below, Fig. 5).
Page 9, lines 16-18 "activation of DYRK1B " and line 38 , "DYRK1B activation" the term "activation" is misleading (elevated expression)
Fig. 3C: (Heatmap) what is the meaning of the white boxes?
Fig. 4A and EV3 (co-IP and co-localization): Taken together, the data provide solid evidence for the interaction of DYRK1B with PB components. Some Figures need improvement and clarification:
Fig. 4A: This experiment looks odd, since both DCP1A and PAT1B are hardly detectable in the control lysates transfected with the empty vector. Is this consistently observed in replicate experiments (it is not seen in the tetOn cell line in panel C)? Are the bands correctly identified (size markers should be indicated)? The DCP1 panel should not be cut so close to the band.
Fig. 4C: In this experiment, DYRK1B inhibition stabilizes the interaction with DYRK1B, which is consistent with a locked-in enzyme substrate complex. However, in Fig. EV3A and B substrate interaction is weaker with the kinase-deficient DYRK1B mutant.
Fig. EV3F: I do not understand the figure. Columns must be labeled. I guess 1st and 4th column show GFP fluorescence and a DNA stain. What exactly is shown in the 2nd and 3rd columns? Same scale as the others? Do the white comet-like structures indicate GFP/magenta and GFP/red colocalization? Would it make sense to show the nuclear dye here? The last column appears to be a magnification, please indicate where it is taken from (as in 4E).
Fig. EV3E: I cannot discriminate effects of DYRK overexpression. Show a blot with better resolution or a Phostag gel or delete.

© 2026. Published by The Company of Biologists under the terms of the Creative Commons Attribution License (https://creativecommons.org/licenses/by/4.0/).

Page 14, line 35: "higher basal abundance of PBs was in part driven by DYRK1B" this experiment does not exclude that endogenous DYRK1A is involved (see the above comment on AZ191 selectivity).

Fig. 5D: "defined as DCP1A and DDX6 positive foci" the legend should state that double positive foci were considered (as in the method section)
panel D (fluorescence microscopy) I am not sure whether I understand this image. Do both images show staining for DCP1A (magenta) and DDX6 (green), i.e. overlays? Only foci were counted, so the non-focal magenta staining is not specific or shows DCP1A outside PB? Please explain in the legend. Why is no DDX6 staining seen in the left panel? I am aware that panel D just shows representative cells to illustrate the procedure, but it may be easier to understand if green and magenta are shown as separate panels of the same cell, or to show ctrl vs siRNA for the same stain.
Page 16 lines 2-7: "Interestingly, we observed an increase in DYRK1B expression in DYRK1A KO cells, perhaps reflecting a compensatory response and some redundant functions for these two kinases" This compensatory increase is important of DYRK1B (but not DYRK1A) has previously been observed, e.g. Ackeifi et al. 2020; JCI Insight 5:e132594, Pastor et al. 2024 PLoS One 19:e0311655).

Fig. 6 (knockdown experiments in PaTu8988T): This experiments convincingly shows the role of DYRK1B as a regulator of PB abundance. However, I am not convinced that a role of DYRK1A can be excluded. The compensatory increase of DYRK1B levels in DYRK1A-ko cells may well conceal the contribution of DYRK1A under normal conditions (see below, discussion section).

Page 18 lines 42-47 (discussion): "This effect was specific to DYRK1B, as DYRK1A did not compensate for its loss. Although DYRK1A phosphorylated PB proteins and localizes to PBs when overexpressed, it did not rescue PB abundance in DYRK1B knockout cells."
It is true that endogenous DYRK1A did not compensate for the loss of DYRK1B, proving a role of DYRK1B as a regulator of PB abundance. However, it cannot be excluded that DYRK1A also contributes to the control of PB, since it was not examined whether ectopic DYRK1A expression could induce phosphorylation of PB proteins (Fig. 2), bind to PB proteins (Fig. 4), and restore PB body abundance in DYRK1B-ko cells (Fig. 6). The BioGRID database contains evidence that several PB body proteins interact with DYRK1A. Notably, several PB components show enhanced phosphorylation in a recent phosphoproteome analysis of DYRK1A overexpressing RPE1 cells (Li et al. 2024 NAR ;52:8897-8912).

Reviewer 2

SUMMARY OF THE ADVANCE MADE IN THIS PAPER AND ITS POTENTIAL SIGNIFICANCE TO THE FIELD

This is a thorough analysis of DYRK1B phosphorylation of the Dcp1a complex. Links to processing bodies characterised. This is an important contribution to the field. Regulation of decapping is observed under various scenarios, but the mechanisms involved had not been well described to date. The manuscript is well written and clear. Thoughtful details are added throughout and limitations discussed.

SUGGESTIONS TO AUTHORS

Minor comments: I think it would add clarity to add molecular weight markers to blots. The migration of some phospho-bands might be indicated with arrows. But no experiments suggested. I would publish as it is.

First revision

Author response to reviewers' comments

Answers to Reviewers:

Reviewer 1:

© 2026. Published by The Company of Biologists under the terms of the Creative Commons Attribution License (https://creativecommons.org/licenses/by/4.0/).

We sincerely thank Reviewer 1 for their constructive assessment and generous comments on the significance of our work. We are grateful that the reviewer found the study clearly presented and valuable for understanding both DYRK1B function and processing body regulation, and we have addressed the specific points raised below.

Minor Comments:

1. Figure 1C (motif analysis) color coding is not obvious and should be explained. We thank the reviewer for this helpful comment. We have amended the Fig. 1C legend to explain the WebLogo colour coding used in the motif analysis. Amino acids are coloured according to their biochemical properties: polar residues are shown in green, basic residues in blue, acidic residues in red, and hydrophobic residues in black. We have also indicated in the legend that the motif analysis was generated using WebLogo (https://weblogo.threeplusone.com).

2. Figure 1D: In contrast to the figure legend, significantly altered phosphopeptides are highlighted in black rather than red. I suggest to highlight the P body proteins. The table underlying the volcano plot should be also be provided as supplemental material to make it easier for readers to evaluate the complete dataset. We thank the reviewer for pointing out this ambiguity. In the original figure legend, the wording incorrectly stated that significantly altered phosphopeptides were highlighted in red. We have revised the Fig. 1D so that it visualizes all the relevant phosphopetides corresponding to P-body proteins (according to GO:0000932) on the volcano plot and amended the legend to clarify that the red shaded region indicates significantly increased phosphopeptides (>1.5-fold, $p<0.05$), while P-body-associated proteins are shown in blue. We have also revised Table S1 to include the phosphopeptide dataset directly underlying the volcano plot as a separate sheet.

3. Table S1: The table should include information about the data presented (metadata, legend to identify the columns) in an extra sheet. This table seems to include both phosphopeptides, unmodified peptides, and those with other modifications. The volcano plot illustrates only phosphopeptides. The table directly underlying the volcano plot could be provided here as an extra sheet (or as an extra file). We agree that the original Table S1 required clearer annotation and that the dataset underlying Fig. 1D should be easier to identify. We have revised Table S1 to include a README_Metadata sheet explaining the dataset, sheet structure, filtering criteria and key analysis columns. The full original peptide level dataset is retained in Table S1_Source sheet. We have also added a separate Volcano Table_Fig1D sheet containing the phosphopeptide dataset used to generate Fig. 1D, thereby distinguishing the volcano plot data from the full Table S1 dataset, which also includes unmodified peptides and peptides with other modifications.

4. Page 6 line 57: "increased phosphorylation of Tyrosine in the activation loop (IYQYIQSR)" Is this rather the increased abundance of this phosphopeptide rather than increased phosphorylation (meaning a ratio of phosphorylated vs unphosphorylated peptide)?- We agree with the reviewer. Since this was a phosphopeptide-enriched dataset and we did not quantify the corresponding unmodified DYRK1B peptide or total DYRK1B abundance in the same analysis, we cannot distinguish increased phosphorylation stoichiometry from increased abundance of the phosphorylated DYRK1B activation-loop peptide. We have therefore revised the text to refer to increased abundance of the DYRK1B phosphopeptide rather than increased phosphorylation.

5. Page 7, lines 43-47: "many of the DYRK1B-driven phosphorylation sites on DCP1A, DCP1B, 4E-T and PAT1B identified in the screen were proline-directed"I could not easily track this information in the data (table S1). column 1 lists the identified peptides, but where is the identification of phosphorylation sites by the MS/MS scans? It would helpful the readers to provide a table of the phosphopeptides relevant to this study with the phosphosites indicated, as well as FC and p values (including SQSTM1 pT269, which is also relevant here, and possibly SF3B1 pT434 as a kind of control for the dataset, given that CDKN1B was not detected). We agree that the phosphosite information should be easier to trace. We have revised Table S1 to include a focused Relevant_Phosphosites_MSMS sheet for the manuscript relevant phosphoproteins, including DCP1A, DCP1B, EIF4ENIF1/4E-T, PATL1/PAT1B, EDC3, EDC4, SQSTM1 and SF3B1. This sheet reports the corresponding Table S1 peptide ID, peptide sequence, fold-change, p-value where available, assigned phosphosite, localisation probability, phosphosite probability scores, proline-directed

© 2026. Published by The Company of Biologists under the terms of the Creative Commons Attribution License (https://creativecommons.org/licenses/by/4.0/).

status and MS/MS evidence identifiers, including best localisation MS/MS ID, raw file and scan number where available. This allows the reader to trace the relevant phosphopeptides and phosphosite assignments more directly.

6. Page 7 line 57: "DYRK1B-selective inhibitor AZ191" the selectivity of AZ191 for DYRK1B over DYRK1A varies in different assays, and at a concentration of 1 µM, DYRK1A is likely also inhibited to some degree. While this is not critical in this experiment with overexpressed DYRK1B, effects on endogenous DYRK1 must be interpreted with caution (see below, Fig. 5). We agree with the reviewer. We have revised the text to avoid implying that AZ191 is fully selective for DYRK1B over DYRK1A at the concentration used. In the relevant sections, we now refer to AZ191 more cautiously as a class I DYRK inhibitor, and we have added text noting that AZ191 may also inhibit DYRK1A at 1 µM.

7. Page 9, lines 16-18 "activation of DYRK1B " and line 38 , "DYRK1B activation" the term "activation" is misleading (elevated expression) We agree that “activation” could be misleading, as DYRK1B was increased by inducible expression. We have therefore replaced “DYRK1B activation” with “DYRK1B expression”

8. Fig. 3C: (Heatmap) what is the meaning of the white boxes? The white boxes represented phosphosites with the highest log2FC values (>6), which were outside the displayed colour scale. This was used to improve visualization of lower magnitude phosphorylation changes. However, we agree that the white colour may be confusing. We have therefore changed these values to the darkest green tone of the scale and clarified this in the figure legend.
Fig. 4A and EV3 (co-IP and co-localization): Taken together, the data provide solid evidence for the interaction of DYRK1B with PB components. Some Figures need improvement and clarification:

9. Fig. 4A: This experiment looks odd, since both DCP1A and PAT1B are hardly detectable in the control lysates transfected with the empty vector. Is this consistently observed in replicate experiments (it is not seen in the tetOn cell line in panel C)? Are the bands correctly identified (size markers should be indicated)? The DCP1 panel should not be cut so close to the band. We agree that Fig. 4A required clearer presentation. Endogenous DCP1A and PAT1B signals in the empty vector transfected input lysates are weak under the exposure conditions used, but the uncropped source blots confirm that both proteins are detected at the expected molecular weights. We have revised the panel to show a less tightly cropped DCP1A blot, added molecular-weight markers, and provide the uncropped blots in the blot transparency part in Supplementary Information.
Fig. 4A and Fig. 4C are not directly comparable, and we have clarified this distinction in the revised legend and Methods. Fig. 4A used transient FLAG-DYRK1B overexpression and whole cell lysates, whereas Fig. 4C used cytosolic lysates from inducible EGFP-DYRK1B stable cells to improve detection of interactions with cytosolic PB components such as DDX6.

10. Fig. 4C: In this experiment, DYRK1B inhibition stabilizes the interaction with DYRK1B, which is consistent with a locked-in enzyme substrate complex. However, in Fig. EV3A and B substrate interaction is weaker with the kinase-deficient DYRK1B mutant. The effects of pharmacological inhibition and kinase-dead mutation may not be directly equivalent. Acute inhibitor treatment may stabilise interactions with an already expressed and folded DYRK1B protein, whereas the kinase-dead D239A mutant may differ in conformation, expression, localisation, or complex assembly. We have therefore softened the interpretation of Fig. 4C, we describe it more cautiously as suggesting that inhibition of DYRK1B activity can alter or stabilise its association with PB components under these conditions.

11. Fig. EV3F: I do not understand the figure. Columns must be labeled. I guess 1st and 4th column show GFP fluorescence and a DNA stain. What exactly is shown in the 2nd and 3rd columns? Same scale as the others? Do the white comet-like structures indicate GFP/magenta and GFP/red colocalization? Would it make sense to show the nuclear dye here? The last column appears to be a magnification, please indicate where it is taken from (as in 4E). This part of Figure EV3 shows that EGFP-tagged DYRK1A and DYRK1B colocalize with the P-body markers DDX6 (magenta) and DCP1A (red), whereas DYRK2 and DYRK3 do not show colocalization with these markers. We thank the reviewer for pointing out the lack of clarity in this figure. To address this, we have revised the figure and added clear column labels. The second and third columns show the additional P-body markers DDX6 (magenta) and DCP1A (red), respectively. The merged images now include boxed

© 2026. Published by The Company of Biologists under the terms of the Creative Commons Attribution License (https://creativecommons.org/licenses/by/4.0/).

regions indicating the areas enlarged in the final "Merged-Zoom" column. These zoomed images highlight representative P-bodies showing colocalization of DDX6, DCP1A, and the corresponding EGFP-tagged DYRK kinase. The plots on the right represent line-scan analyses across the enlarged P-bodies, demonstrating the spatial overlap of the fluorescence signals. We hope these modifications will improve the clarity and interpretation of the figure.

12. Fig. EV3E: I cannot discriminate effects of DYRK overexpression. Show a blot with better resolution or a Phostag gel or delete. We agree that the original Fig. EV3E did not allow clear discrimination of all DYRK-dependent effects. We have increased the size and resolution of the panel and changed the legend to avoid overinterpreting the blot. The revised text now focuses on the modest mobility changes in DCP1A and 4E-T upon DYRK1A and DYRK1B overexpression. We also note separately that Fig. EV3F shows the clearest co-localisation of DYRK1A and DYRK1B with PB markers in HeLa cells. The Result section has also been amended to present these observations more cautiously and to avoid excluding possible contributions from other DYRK family members.

13. Page 14, line 35: "higher basal abundance of PBs was in part driven by DYRK1B" this experiment does not exclude that endogenous DYRK1A is involved (see the above comment on AZ191 selectivity). We agree with the reviewer that AZ191 treatment at 1 μM cannot be interpreted as exclusively inhibiting DYRK1B, and therefore does not exclude a contribution from endogenous DYRK1A. We have revised the text to avoid this overinterpretation. The AZ191 data are now described as evidence that class I DYRK kinase activity contributes to PB abundance, while the DYRK1B specific conclusion is based on the genetic approaches, including DYRK1B siRNA knockdown and DYRK1B knockout and rescue experiments

14. Fig. 5D: "defined as DCP1A and DDX6 positive foci" the legend should state that double positive foci were considered (as in the method section)panel D (fluorescence microscopy) I am not sure whether I understand this image. Do both images show staining for DCP1A (magenta) and DDX6 (green), i.e. overlays? Only foci were counted, so the non-focal magenta staining is not specific or shows DCP1A outside PB? Please explain in the legend. Why is no DDX6 staining seen in the left panel? I am aware that panel D just shows representative cells to illustrate the procedure, but it may be easier to understand if green and magenta are shown as separate panels of the same cell, or to show ctrl vs siRNA for the same stain. We agree that the original presentation of Fig. 5D did not make the scoring criteria sufficiently clear. We have revised the legend to state explicitly that only discrete cytoplasmic foci positive for both DCP1A and DDX6 were counted as PBs, as described in the Methods. To improve interpretation, we have revised panel D to show the DCP1A and DDX6 channels separately, together with the merged image, so that the loss of DCP1A and DDX6 double positive PB foci after DYRK1B depletion is clearer.

15. Page 16 lines 2-7: "Interestingly, we observed an increase in DYRK1B expression in DYRK1A KO cells, perhaps reflecting a compensatory response and some redundant functions for these two kinases" This compensatory increase is important of DYRK1B (but not DYRK1A) has previously been observed, e.g. Ackeifi et al. 2020; JCI Insight 5:e132594, Pastor et al. 2024 PLoS One 19:e0311655). We thank the reviewer for pointing this out. We have revised the text to cite previous reports showing compensatory upregulation of DYRK1B following DYRK1A loss or inhibition.

16. Fig. 6 (knockdown experiments in PaTu8988T): This experiments convincingly shows the role of DYRK1B as a regulator of PB abundance. However, I am not convinced that a role of DYRK1A can be excluded. The compensatory increase of DYRK1B levels in DYRK1A-ko cells may well conceal the contribution of DYRK1A under normal conditions (see below, discussion section).

Page 18 lines 42-47 (discussion): "This effect was specific to DYRK1B, as DYRK1A did not compensate for its loss. Although DYRK1A phosphorylated PB proteins and localizes to PBs when overexpressed, it did not rescue PB abundance in DYRK1B knockout cells."It is true that endogenous DYRK1A did not compensate for the loss of DYRK1B, proving a role of DYRK1B as a regulator of PB abundance. However, it cannot be excluded that DYRK1A also contributes to the control of PB, since it was not examined whether ectopic DYRK1A expression could induce phosphorylation of PB proteins (Fig. 2), bind to PB proteins (Fig. 4), and restore PB body abundance in DYRK1B-ko cells (Fig. 6). The BioGRID database contains evidence that several PB body proteins interact with DYRK1A. Notably, several PB components show enhanced phosphorylation in a recent phosphoproteome analysis of DYRK1A overexpressing RPE1 cells (Li et al. 2024 NAR ;52:8897-8912).

© 2026. Published by The Company of Biologists under the terms of the Creative Commons Attribution License (https://creativecommons.org/licenses/by/4.0/).

We agree with the reviewer that our data do not exclude a contribution of DYRK1A to PB regulation. We have therefore revised the Results and Discussion to avoid stating that the effect is specific to DYRK1B or that DYRK1A has no role in PB biology. We now state that DYRK1A knockout did not reduce PB abundance under the conditions tested, but that increased DYRK1B expression in DYRK1A KO cells could mask a contribution from DYRK1A. However, to our knowledge, previous studies have not directly demonstrated regulation of PB abundance or PB homeostasis by DYRK kinases. We therefore present our study as establishing DYRK1B as a regulator of PB abundance, while noting that DYRK1A may also contribute to PB regulation in other cellular contexts by citing Li et al. 2024 NAR ;52:8897-8912 as suggested by the reviewer.

Reviewer2:

Minor comments: I think it would add clarity to add molecular weight markers to blots. The migration of some phospho-bands might be indicated with arrows. But no experiments suggested. I would publish as it is.

We thank Reviewer 2 for these encouraging comments and helpful suggestions. We have revised the relevant immunoblots to improve clarity by adding molecular weight markers to all gels. The exceptions are Figure 2C and Figure 3B which are Phos-Tag gels; it is well known that molecular weight marker ladders do not run true on Phos-tag gels (Nagi Z et al., Current Protocols, 2018). We have also replaced some representative blot images with clearer replicates in which the phosphorylation dependent mobility shifts are more evident (Fig2).

Second decision letter

MS ID#: jcs.265054R1

MS Title: Phosphoproteomics identifies the DYRK1B protein kinase as a regulator of processing bodies

Authors: Anne L Ashford; Suzan Ber; Miriam S Ems; Emma Duncan; Kathryn Balmanno; Hannah Reeves; Rachael Huntly; Megan A Cassidy; Harvey E Johnston; David Oxley; Thaddeus M Nthiga; Terje Johansen; Marie Kluge; Ralf Jacob; Matthias Lauth; Simon J Cook

Article Type: Research Article

Dear Dr Cook,

I am happy to tell you that your manuscript has been accepted for publication in Journal of Cell Science, pending standard publication integrity checks.

© 2026. Published by The Company of Biologists under the terms of the Creative Commons Attribution License (https://creativecommons.org/licenses/by/4.0/).
